# Global Transcriptional Repression of Diguanylate Cyclases by MucR1 Is Essential for *Sinorhizobium*-Soybean Symbiosis

**DOI:** 10.1128/mBio.01192-21

**Published:** 2021-10-26

**Authors:** Meng-Lin Li, Jian Jiao, Biliang Zhang, Wen-Tao Shi, Wen-Hao Yu, Chang-Fu Tian

**Affiliations:** a State Key Laboratory of Agrobiotechnology, MOA Key Laboratory of Soil Microbiology, and Rhizobium Research Center, College of Biological Sciences, China Agricultural University, Beijing, China; University of Nebraska-Lincoln

**Keywords:** c-di-GMP, diguanylate cyclase, nodule, rhizobia

## Abstract

The ubiquitous bacterial second messenger c-di-GMP is intensively studied in pathogens but less so in mutualistic bacteria. Here, we report a genome-wide investigation of functional diguanylate cyclases (DGCs) synthesizing c-di-GMP from two molecules of GTP in Sinorhizobium fredii CCBAU45436, a facultative microsymbiont fixing nitrogen in nodules of diverse legumes, including soybean. Among 25 proteins harboring a putative GGDEF domain catalyzing the biosynthesis of c-di-GMP, eight functional DGCs were identified by heterogenous expression in Escherichia coli in a Congo red binding assay. This screening result was further verified by *in vitro* enzymatic assay with purified full proteins or the GGDEF domains from representative functional and nonfunctional DGCs. In the same *in vitro* assay, a functional EAL domain catalyzing the degradation of c-di-GMP into pGpG was identified in a protein that has an inactive GGDEF domain but with an active phosphodiesterase (PDE) function. The identified functional DGCs generally exhibited low transcription levels in soybean nodules compared to free-living cultures, as revealed in transcriptomes. An engineered upregulation of a functional DGC in nodules led to a significant increase of c-di-GMP level and symbiotic defects, which were not observed when a functional EAL domain was upregulated at the same level. Further transcriptional analysis and gel shift assay demonstrated that these functional DGCs were all transcriptionally repressed in nodules by a global pleiotropic regulator, MucR1, that is essential in *Sinorhizobium*-soybean symbiosis. These findings shed novel insights onto the systematic regulation of c-di-GMP biosynthesis in mutualistic symbiosis.

## INTRODUCTION

Cyclic dimeric GMP (c-di-GMP) is a ubiquitous second messenger in bacteria, regulating key functions and mechanisms such as biofilm formation, transition from motility to sessility, cell cycle, and differentiation (1). Most of these pathways are involved in bacterial interactions with abiotic surfaces or with other bacterial and eukaryotic cells (1, 2). c-di-GMP is a diffusible intracellular molecule synthesized from two GTP molecules by diguanylate cyclases (DGCs) containing the GGDEF domain and can be degraded into 5′-phosphoguanylyl-(3′–5′)-guanosine (pGpG) and/or two GMP molecules by phosphodiesterases (PDEs) bearing the EAL or HD-GYP domains (3, 4). c-di-GMP homeostasis is modulated by DGCs and PDEs and can be sensed by effectors, including the PilZ domain, GIL domain, MshEN domain, riboswitch, transcriptional factors, and degenerate GGDEF or EAL domain (4, 5). The high diversity in DGCs, PDEs, and c-di-GMP effectors and their subsequent regulation accounts for the multiple roles of c-di-GMP in bacterial adaptations to fluctuating abiotic and biotic conditions.

c-di-GMP signaling has been mainly and intensively studied in bacterial pathogens but less so in mutualistic bacteria (2, 6–12). As model mutualistic microsymbionts, rhizobia induce and intracellularly infect root nodules, where they fix atmospheric N_2_ into ammonia to support legume growth (13). The energy-consuming process of rhizobial nitrogen fixation is sustained by nutrients provided by host cells (14). Rhizobia can live saprophytically in soils in the absence of a compatible legume host and represent a typical facultative microsymbiont bearing larger genomes than those obligate microsymbionts to cope with fluctuating stimuli (15). Constitutive expression of a DGC (PleD) from *Caulobacter crescentus* in Rhizobium etli and Rhizobium leguminosarum strains favored exopolysaccharide (EPS) production and adhesion to legume roots but decreased the fresh weight of inoculated plants (9). A similar constitutive expression of PleD from C. crescentus in Sinorhizobium meliloti allowed the discovery of cryptic EPSs such as a linear mixed-linkage beta-glucan and an arabinose-containing polysaccharide (6, 7, 12), although the plasmid carrying this heterologous PleD was lost rapidly under nonselective conditions, including the rhizosphere of the legume host alfalfa (7). A later study showed that an S. meliloti mutant lacking 16 out of 17 GGDEF-encoding genes had no salient phenotypes under tested free-living and symbiotic conditions except for its decreased tolerance to acid stress (11), although this strain had no detectable c-di-GMP under test conditions. In contrast, a c-di-GMP-free derivative of C. crescentus showed severe defects in its bimodal life cycle, motility, and surface attachment (16), and a Salmonella enterica serovar Typhimurium mutant lacking all GGDEF-encoding genes lost virulence and exhibited various defects in free-living processes, such as motility, biofilm formation, and cellulose biosynthesis (17). In short, despite open pangenomes of rhizobial species and dozens of c-di-GMP signaling components in individual genomes (10, 18), c-di-GMP signaling in rhizobia remains largely unexplored.

In this work, we focused on Sinorhizobium fredii CCBAU45436 (SF45436), which can establish effective symbiosis with soybean and many other legumes (19, 20). A genome-wide bioinformatic analysis of proteins with putative GGDEF, EAL, and HD-GYP domains was performed. The functional DGCs were screened by heterogenous expression of the full proteins or GGDEF domain alone in Escherichia coli in a canonical Congo red binding assay. Enzymatic activities of DGC or PDE were further tested for purified full proteins, GGDEF or EAL domains from representative functional, and nonfunctional DGCs. Transcriptome sequencing (RNA-seq) was used to determine transcriptional profiles of DGCs at exponential and stationary phases in free-living culture and in soybean nodules. The potential role of c-di-GMP in symbiosis was tested by using deletion mutants of major DGCs transcribed in soybean nodules or engineered strains harboring nodule-specific overexpression of a functional DGC or an active EAL domain. Finally, we studied the direct transcriptional regulation of functional DGCs by a pleotropic regulator, MucR, which modulates various canonical processes responding to c-di-GMP (21) and is essential for symbiotic efficiency of SF45436 on soybean (22).

## RESULTS AND DISCUSSION

### Screening functional diguanylate cyclases in Sinorhizobium fredii SF45436.

**FIG 1:**
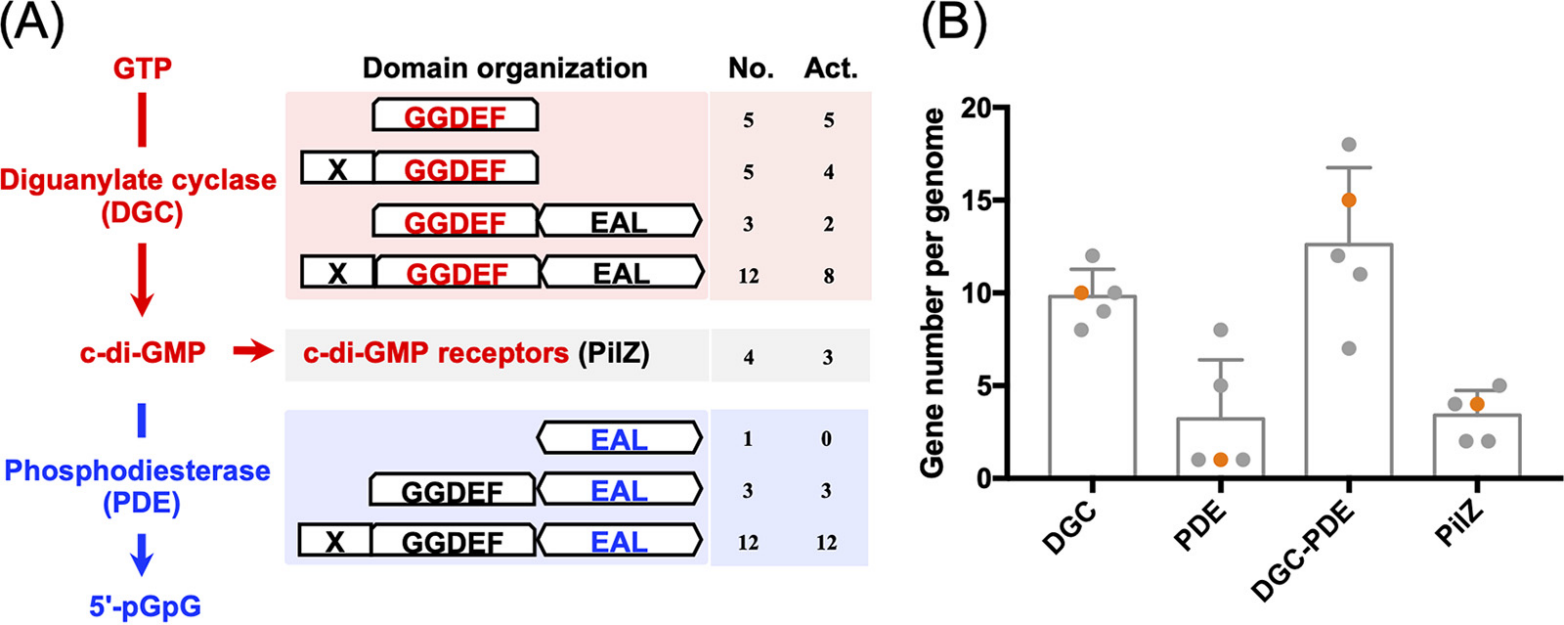
Overview of c-di-GMP signaling in Sinorhizobium fredii SF45436. (A) Synthesis of c-di-GMP by diguanylate cyclase (DGC) harboring the GGDEF domain and degradation of c-di-GMP by phosphodiesterase (PDE) containing the EAL domain. X indicates various sensory domains. The number of proteins, with each corresponding domain organization form, encoded by the SF45436 genome is shown (No.). Among them, the number of proteins with a conserved active motif [D_X(7 aa)_N_X(8 aa)_D_X(21 aa)_R_X_G_/S/A_GD_/E_EF] is indicated (Act). (B) The number of genes encoding proteins with putative activity of DGC or PDE, DGC-PDE bifunctional proteins, or PilZ-like proteins in *Sinorhizobium* sibling species. The point in orange corresponds to the value of SF45436. The other test *Sinorhizobium* strains include *S. fredii* SF25509, *S. fredii* SF83666, *S. sojae* SJ05684, and *Sinorhizobium* sp. strain SS05631. Error bars represent SD.

Genome-wide analysis of *S. fredii* SF45436 uncovered 25 GGDEF-containing proteins, which show various domain organizations (Fig. 1A). Putative receptors of c-di-GMP and phosphodiesterases (PDE) were also identified, indicating that complete c-di-GMP signaling may exist in SF45436. Similar numbers of c-di-GMP signaling components are in complete genomes of other *Sinorhizobium* strains associated with soybeans (23, 24), such as *S. fredii* SF25509 (26 proteins), *S. fredii* SF83666 (35 proteins), *S. sojae* SJ05684 (26 proteins), and *Sinorhizobium* sp. strain SS05631 (28 proteins) (Fig. 1B).

**FIG 2:**
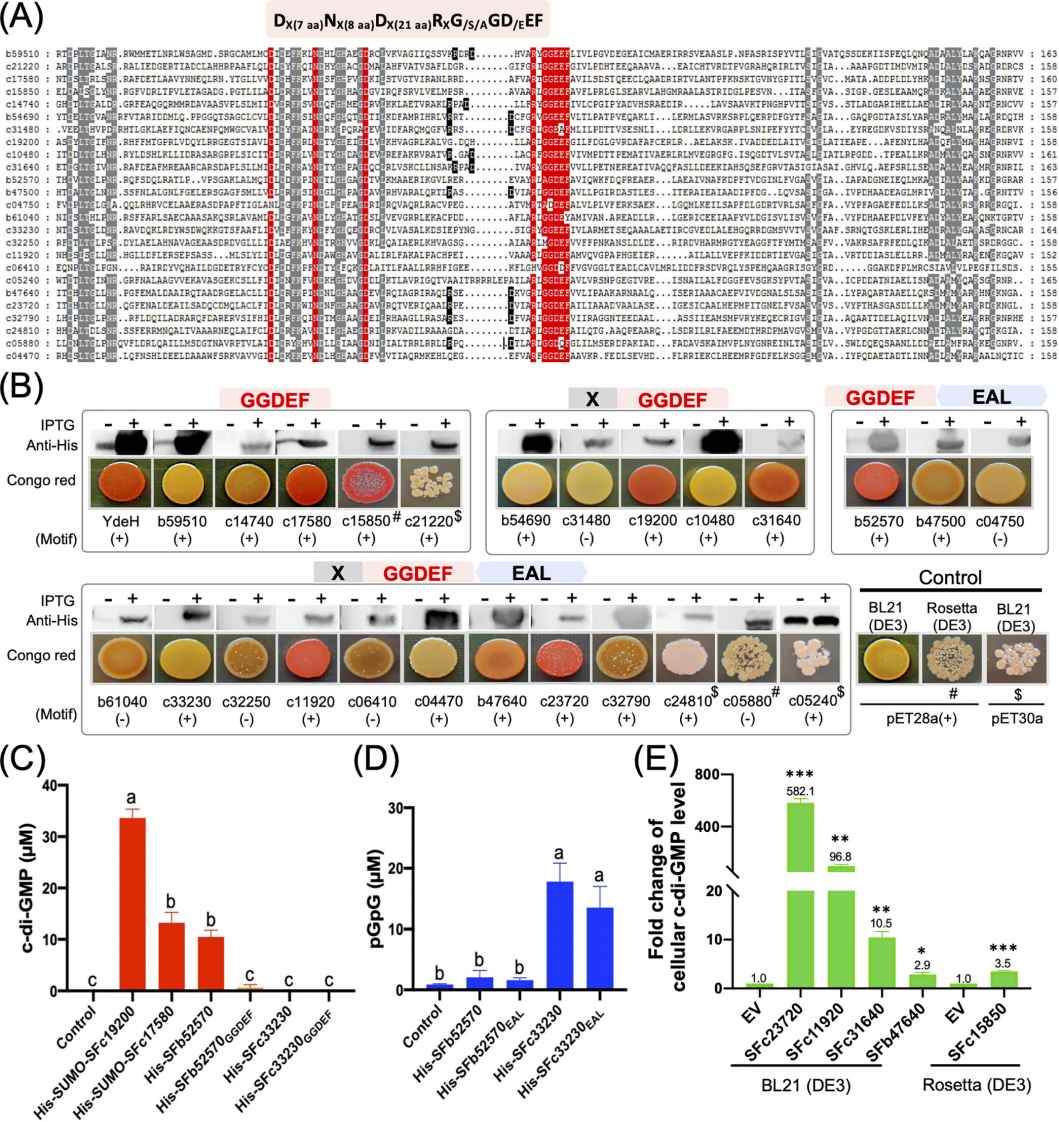
Characterization of the GGDEF-containing proteins from *S. fredii* SF45436. (A) A sequence alignment of the GGDEF domain. A reported conserved motif essential for functional DGCs is shown. Gray background indicates other conserved amino acids in this sequence alignment (present in more than 80% sequences). Residues in black background form the reported allosteric I site involved in product inhibition of c-di-GMP synthesis. (B) Congo red binding ability of E. coli strains harboring various GGDEF-containing proteins from SF45436. Western blotting with anti-His monoclonal antibody shows the induced expression of corresponding proteins by IPTG. (+) and (−) indicate proteins with/without a conserved active motif [D_X(7 aa)_N_X(8 aa)_D_X(21 aa)_R_X_G_/S/A_GD_/E_EF]. YdeH is a known functional DGC from E. coli. For proteins not expressed in BL21(DE3) harboring pET28a(+) derivatives, the Rosetta strains (#) containing the same expressing plasmids or BL21(DE3) carrying pET30a derivatives ($) were tested. (C and D) HPLC-MS determination of c-di-GMP (C) or pGpG (D) content in 1 μM purified protein prepared from E. coli strains harboring corresponding vectors derived from pET30a or pET28a(+). Error bars represent standard deviations. Different letters above error bars indicate significant difference between means based on three independent experiments (ANOVA followed by Bonferroni’s multiple-comparison test, α = 0.05). (E) HPLC-MS determination of c-di-GMP content in E. coli strains expressing the indicated proteins relative to that of strains carrying the empty vector (*, *P < *0.05; **, *P < *0.01; ***, *P < *0.001; *t* test). Error bars represent SD. Protein purification for these five proteins was not successful under test conditions, and *in vitro* enzymatic assay was not done.

Among those GGDEF-containing proteins in SF45436 (Fig. 2A), 19 of them have GGDEF domains of a conserved motif [D_X(7 aa)_N_X(8 aa)_D_X(21 aa)_R_X_G_/S/A_GD_/E_EF] reported in functional diguanylate cyclases (1). In E. coli, the c-di-GMP biosynthesis mediated by a functional diguanylate cyclase can be indicated by the biosynthesis of cellulose with strong Congo red binding ability (25). To verify the activity of putative diguanylate cyclases in SF45436, Congo red binding ability of E. coli strains expressing individual GGDEF-containing proteins from SF45436 was tested (Fig. 2B). Western blot analysis showed notable induced expression by isopropyl-β-d-thiogalactopyranoside (IPTG) for individual proteins in E. coli BL21 or Rosetta, although leak expression was observed for those strains harboring *ydeH*, *SFb59510*, *SFc17580*, and *SFc05240* (Fig. 2B). Similar to the known diguanylate cyclase YdeH from E. coli (26), overexpressing SFc17580, SFc15850, SFc19200, SFc31640, SFb52570, SFc11920, SFc23720, and SFb47640 from SF45436 was able to enhance the ability of engineered E. coli cells to bind Congo red. All eight of these proteins and YdeH have the conserved motif [D_X(7 aa)_N_X(8 aa)_D_X(21 aa)_R_X_G_/S/A_GD_/E_EF], while overexpression of some other proteins with this motif could not enable E. coli to bind Congo red (Fig. 2B).

Diverse N-terminal domains, such as REC-REC (SFc19200), 5TM-5TMR_LYT-PAS_4-PAS_7 (SFc31640), CHASE4 (SFc11920), and HAMP-PAS (SFb47640 and SFc23720), are associated with GGDEF in these functional DGCs, implying their potential roles in directly sensing fluctuating stimuli or interacting with other proteins in the life cycle of this facultative microsymbiont of various legumes (19, 20, 27). For example, diverse PAS domains can serve as direct sensors of various ligands, including oxygen, blue light, cellular redox, carboxylate-containing substrates, divalent metal, and fatty acid (28). DGCs with the REC domain are responsive regulators of two-component signal transduction systems, responding to extracellular or intracellular signals perceived by their cognate sensor His kinases (1). When only the GGDEF domain from 20 proteins of multiple domains was overexpressed in E. coli, none of them was functional (see Fig. S1 in the supplemental material). Since stable single GGDEF-domain-bearing DGCs are not rare (Fig. 2B), this implies potential misfolding of the cloned GGDEF domain from SFc19200, SFc31640, SFb52570, SFc11920, SFc23720, and SFb47640. This view was further supported by high-performance liquid chromatography mass spectrometry (HPLC-MS) analysis of c-di-GMP production using purified His-SFb52570_GGDEF_ and His-SFb52570 (Fig. S2) in the presence of GTP, i.e., the full protein of SFb52570 rather than SFb52570_GGDEF_ alone had diguanylate cyclase activity (Fig. 2C). Moreover, phosphodiesterase activity was not detectable for His-SFb52570_EAL_ and His-SFb52570, suggesting a degenerated EAL domain in this protein (Fig. 2D). The degenerate EAL domain of SFb52570 may play a structural or regulatory function, as deletion of this enzymatically inactive EAL domain abolished the DGC activity of SFb52570 (Fig. 2C, Fig. S1). Similarly, DGCs with degenerate EAL domains are also experimentally demonstrated in Gluconacetobacter xylinus (29, 30).

10.1128/mBio.01192-21.1 FIG S1 Congo red binding ability of E. coli strains harboring various GGDEF domains from corresponding proteins of SF45436. YdeH is a known functional DGC from E. coli. Download FIG S1, PDF file, 0.7 MB. Copyright © 2021 Li et al. 2021 Li et al. https://creativecommons.org/licenses/by/4.0/ This content is distributed under the terms of the Creative Commons Attribution 4.0 International license.

10.1128/mBio.01192-21.2 FIG S2 SDS-PAGE gels of purified proteins. His-SFc19200, His-SFc17580, and His-MucR1 were poorly soluble *in vitro*, and the His-SUMO versions were purified. Download FIG S2, PDF file, 0.1 MB. Copyright © 2021 Li et al. 2021 Li et al. https://creativecommons.org/licenses/by/4.0/ This content is distributed under the terms of the Creative Commons Attribution 4.0 International license.

Both purified His-SFc33230 and His-SFc33230_EAL_ (Fig. S2) exhibited phosphodiesterase activity generating 5′-pGpG from c-di-GMP, whereas no diguanylate cyclase activity was detected for His-SFc33230 and His-SFc33230_GGDEF_ (Fig. 2D and C). The purified His-SUMO-SFc17580 with only the GGDEF domain and His-SUMO-SFc19200 (Fig. S2; His-SFc17580 and His-SFc19200 were poorly soluble) were able to produce c-di-GMP from GTP (Fig. 2C). Although protein purification for the other five functional DGCs (SFc23720, SFc11920, SFc31640, SFb47640, and SFc15850) was not successful under test conditions, HPLC-MS analysis revealed significantly higher levels of c-di-GMP in E. coli strains expressing these five DGCs than in strains carrying empty vectors (Fig. 2E; *P*  < 0.05, *t* test). These results are consistent with those of Congo red binding assay.

### Transcriptional profiles of functional diguanylate cyclases and the effect of c-di-GMP elevation in soybean nodules.

**FIG 3:**
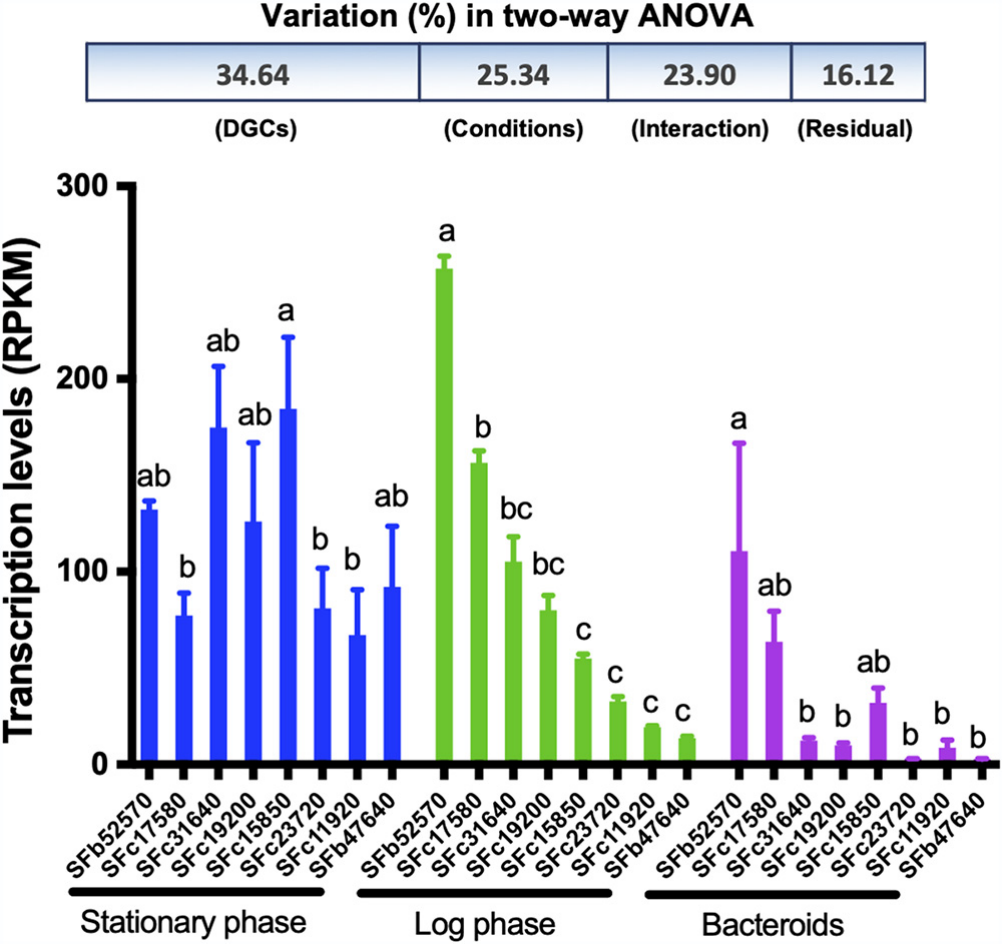
Transcription profiles of functional diguanylate cyclases in free-living and symbiotic SF45436. Different letters indicate significant differences between means of three biological replicates (two-way ANOVA followed by Bonferroni’s multiple-comparison test under each condition; α = 0.05). RPKM, reads per kilobase per million mapped reads. Error bars represent SEM.

RNA-seq analysis revealed that eight functional diguanylate cyclases exhibited contrasting transcriptional profiles during exponential (log) and stationary phases and within soybean nodules (Fig. 3). The largest proportion of total variation (34.64%) was explained by different diguanylate cyclases, followed by different conditions (25.34%) and interaction between these two main effects (23.9%). SFb52570 and SFc17580 were major diguanylate cyclases transcribed during the log phase and within symbiotic nodules (Fig. 3), although all functional DGCs were actively transcribed at stationary phase, suggesting that multiple stress signals arise under this nutrient-starving condition. In line with these findings, two DGCs had relatively strong expression during all growth phases of E. coli, while a number of DGCs were induced during stationary phase (25).

**FIG 4:**
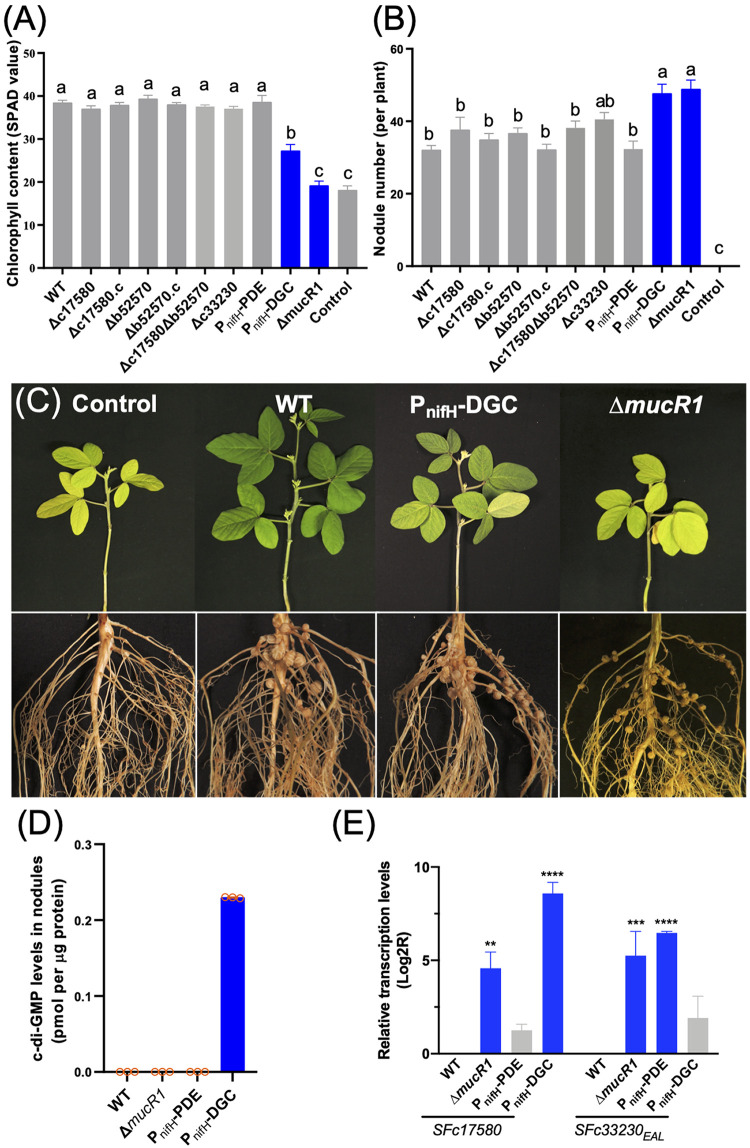
Symbiotic defects mediated by c-di-GMP overload in soybean nodules. (A) Chlorophyll content. (B) Nodule number per plant. Test strains include deletion (Δ) or complementary (.c) strains for DGC genes (*SFc17580* and *SFb52570*), the deletion mutant of PDE gene (*SFc33230*), and derivatives carrying PDE (*SFc33230_EAL_*) and DGC (*SFc17580*) driven by the *nifH* (*SFa46030*) promoter P_nifH_. The Δ*mucR1* mutant was used for comparison. Error bars represent SEM. Different letters indicate significant differences between means based on more than 12 plants (ANOVA followed by Turkey’s multiple-comparison test; α = 0.05). (C) Pictures indicating yellow leaves and the increased nodule number of soybean plants inoculated with the P_nifH_-DGC and the Δ*mucR1* mutant. (D) c-di-GMP levels in nodules (three biological replicates; error bars represent SD). (E) Transcriptional changes of *SFc17580* and *SFc33230_EAL_* in test strains (three biological replicates, ANOVA followed by Dunnett’s multiple comparisons; ***, *P* < 0.001; ****, *P* < 0.0001; error bars represent SD).

In-frame deletion of either *SFb52570* (**Δ***b52570*) or *SFc17580* (**Δ***c17580*) or both major DGC genes (**Δ***c17580* **Δ***b52570*) had no significant symbiotic defects on soybean plants (Fig. 4A and B). In contrast, a derivative carrying the functional DGC SFc17580 (Fig. 2C) driven by the *nifH* promoter (P_nifH_-DGC) induced more but inefficient nodules, leading to a significant decline in chlorophyll content of soybean leaves (Fig. 4A to C). Since *nifH* encodes the nitrogenase reductase and is specifically expressed in nodules induced by most rhizobia without free-living nitrogen fixation ability (22, 31), the impaired symbiotic performance of the P_nifH_-DGC strain is strictly nodule specific. This is in contrast to earlier studies of *R. etli* and R. leguminosarum using a constitutive expression version of heterogenous PleD from C. crescentus, which showed a decline in both nodule number and nitrogen content of corresponding host plants Phaseolus vulgaris and *Vicia sativa* (9). When the transcription of the functional EAL domain of SFc33230 (Fig. 2D) was driven by P_nifH_ (P_nifH_-PDE), no symbiotic defects were observed on soybean plants (Fig. 4A and B). This is consistent with the normal phenotype of alfalfa plants inoculated with an S. meliloti mutant lacking 16 out of 17 GGDEF-encoding genes and having no detectable c-di-GMP (11).

The HPLC-MS analysis (Fig. 4D) further showed that c-di-GMP was at the level of pmol per microgram protein in nodules infected by the PnifH-DGC strain but undetectable in the other treatments (the wild-type SF45436, **Δ***mucR1*, and P_nifH_-PDE strains). Further quantitative reverse transcription-PCR (qRT-PCR) analysis (Fig. 4E) revealed that the DGC gene *SFc17580* and the EAL-encoding fragment *SFc33230_EAL_* were actively transcribed in the P_nifH_-DGC and P_nifH_-PDE strains, respectively. This demonstrated the efficiency of the test P_nifH_ promoter in nodules, and the high expression of *SFc33230_EAL_* had no significant effect on symbiosis. In short, elevating rhizobial c-di-GMP in nodules exerted a negative effect on symbiotic performance.

For comparison, the in-frame deletion mutant of the functional PDE gene *SFc33230* (Fig. 2D) or the pleotropic regulator gene *mucR1* (*mucR2*, the other *mucR* copy in SF45436, is not functional due to a frameshift mutation) (21, 22) were also tested for their symbiotic performance. As many as 31 EAL domain-containing proteins are present in SF45436 (Fig. 1A); consequently, it is not unexpected that **Δ***c33230* strain was indistinguishable from the wild-type SF45436 in symbiotic performance (Fig. 4A and B). The **Δ***mucR1* mutant showed more severe symbiotic defects than the P_nifH_-DGC strain regarding the chlorophyll content of leaves (Fig. 4A and C), although it induced as many nodules as the P_nifH_-DGC strain (Fig. 4B and C). Both *SFc17580* and *SFc33230_EAL_* were upregulated in the **Δ***mucR1* mutant (Fig. 4E). This may at least partially explain the undetectable c-di-GMP in nodules infected by the **Δ***mucR1* mutant, implying an intriguing regulation role of MucR1 on c-di-GMP signaling components.

**FIG 5:**
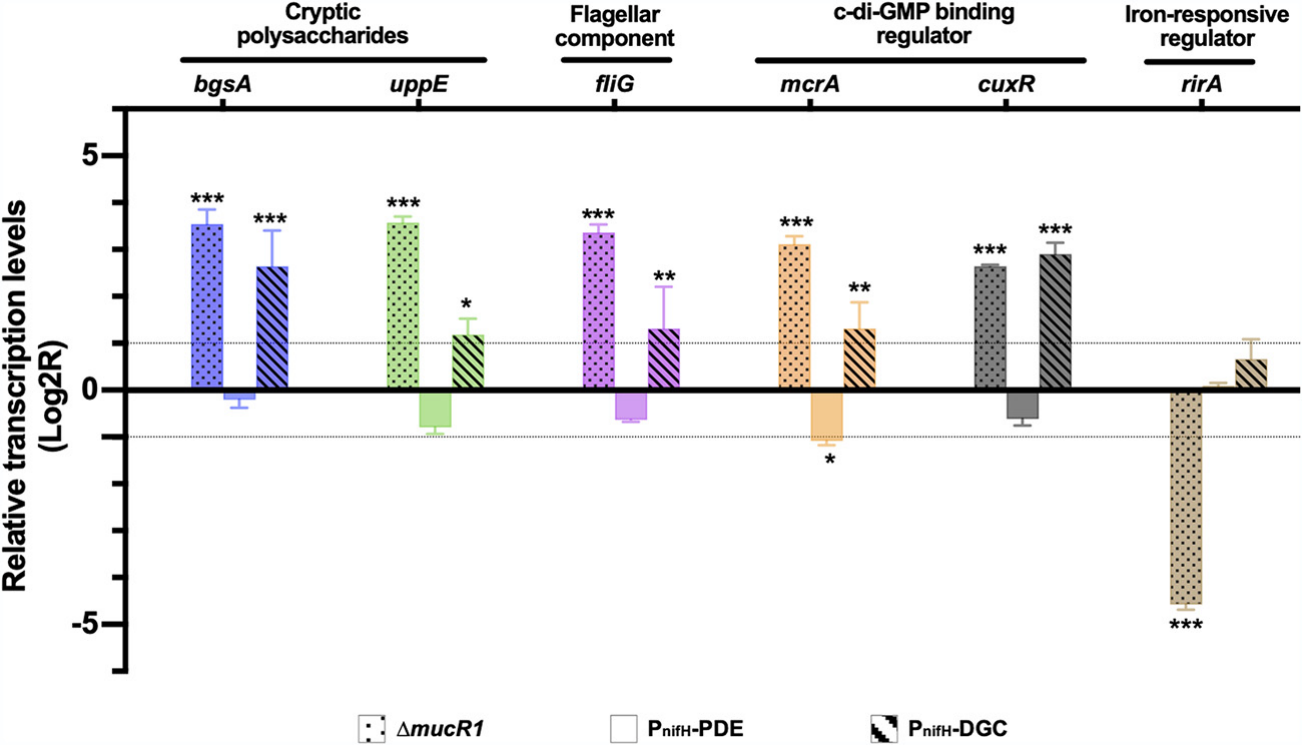
Transcriptional changes of c-di-GMP responsive genes of the Δ*mucR* mutant and the P_nifH_-DGC and P_nifH_-PDE strains in soybean nodules. An iron-responsive regulator gene, *rirA*, is used as a control. Significant differences compared to the wild-type SF45436 are indicated (three biological replicates, ANOVA followed by Dunnett’s multiple comparisons; **, *P* < 0.01; ***, *P* < 0.001).

Moreover, bacteroids of the P_nifH_-DGC strain and the **Δ***mucR1* mutant but not those of the P_nifH_-PDE strain showed significant upregulation of genes involved in various c-di-GMP-responsive processes (Fig. 5), such as the c-di-GMP receptor McrA, regulating motility (11), and CuxR, activating arabinose-containing polysaccharide production (6), the biosynthesis of mixed-linkage beta-glucan (*bgsA*) and adhesion polysaccharides (*uppE*), and a key flagellar component (*fliG*) (12, 32, 33). Notably, both polysaccharides and motility machinery have energetic cost in bacterial physiology and are downregulated during nitrogen fixation that consumes as many as 16 ATP to reduce one molecule of N_2_ (22, 27, 34). This is supported by recent evidence that optimum energy metabolism status of bacteroids is required for efficient symbiosis in soybean nodules (24).

Since no c-di-GMP could be detected in bacteroids of the **Δ***mucR1* mutant while the P_nifH_-DGC strain significantly accumulated c-di-GMP in nodules (Fig. 4E), the transcription of the c-di-GMP-responsive processes mentioned above may be subject to a general negative regulation by MucR1, which can be relieved to a certain extent by elevating c-di-GMP levels (Fig. 5). Notably, MucR1 can also act as a positive regulator required for active transcription of high-affinity transporters for phosphate and zinc ions and the iron-responsive regulator RirA, which are all essential for efficient symbiosis of *S. fredii* within soybean nodules (22, 35–38). The downregulation of *rirA* in bacteroids of the *mucR1* mutant was confirmed in this work but not observed in bacteroids of the P_nifH_-DGC strain (Fig. 5). This may partially explain the more severe symbiotic defects associated with the **Δ***mucR1* mutant than the P_nifH_-DGC strain (Fig. 4A to C).

### Direct regulation of functional DGCs by MucR1.

**FIG 6:**
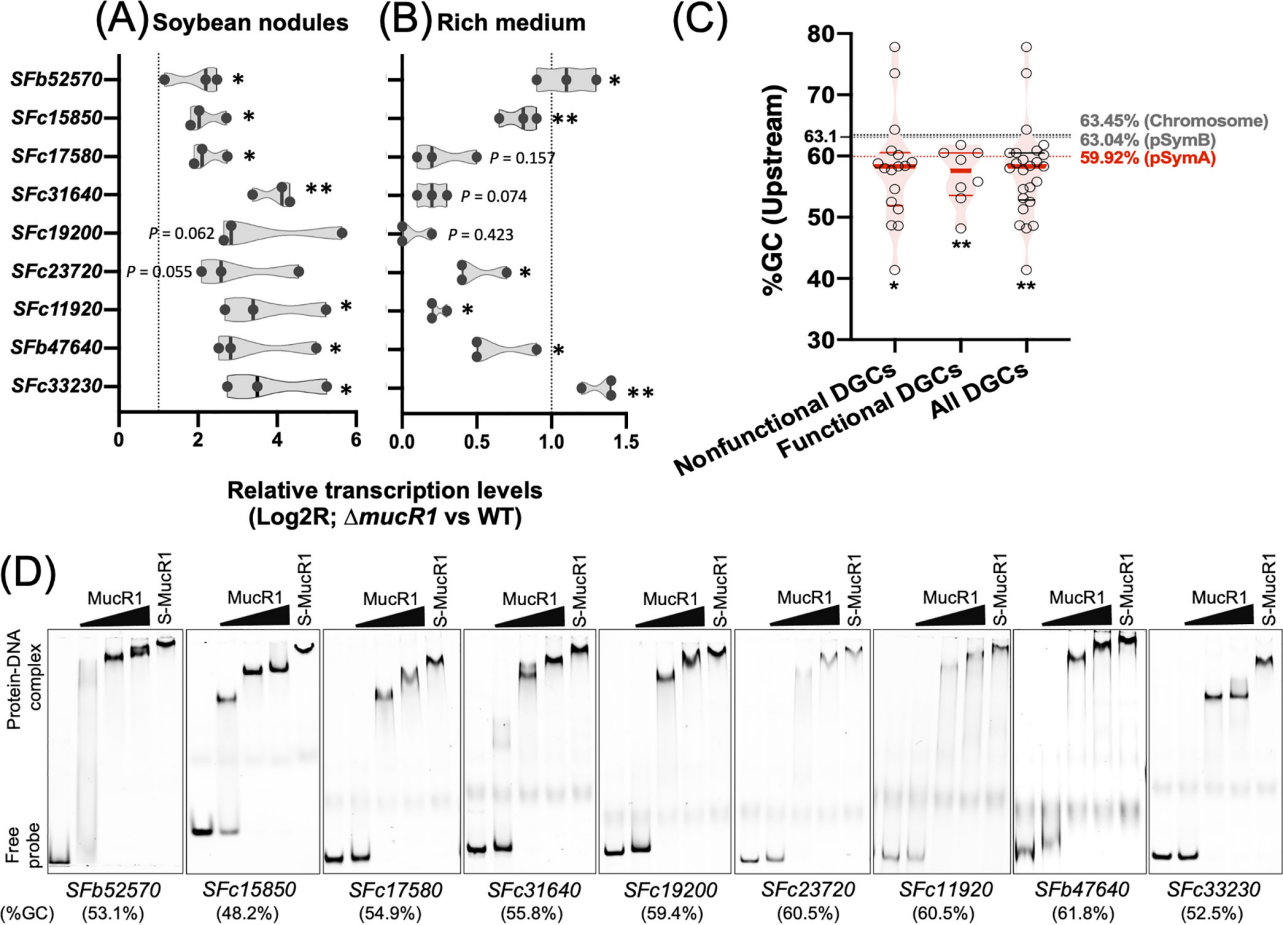
Direct transcriptional repression of eight functional DGCs and the PDE SFc33230 by MucR1 in soybean nodules. (A and B) Transcriptional changes of functional DGC genes in the Δ*mucR1* mutant compared to the wild-type SF45436 in soybean nodules (A) and the TY medium at an OD_600_ of 1.2 (B), based on qRT-PCR (three biological replicates; *, *P < *0.05; **, *P < *0.01; *t* test). (C) Average %GC of upstream intergenic region for genes encoding functional or nonfunctional DGCs. The genome average of 63.1% and those average values of three major replicons are shown. Significant difference compared to the genome average value is indicated (*, *P* < 0.05; **, *P* < 0.01; *t* test). (D) MucR1 binds the promoters of genes encoding eight functional DGCs and the PDE SFc33230 in the electrophoretic mobility shift assay (EMSA). The purified Sumo-MucR1 (27 μM, S-MucR1) and its derivative, MucR1 (Sumo was removed by HRV-3C protease), with increasing concentrations (4.5, 13.5, and 27 μM) were incubated with Cy5-labeled DNA probes. %GC of test probes are shown.

The previous transcriptomic analyses in alphaproteobacteria have revealed various processes responding to c-di-GMP, and some GGDEF/EAL-encoding genes are differentially transcribed when *mucR1* is mutated (21, 22). As mentioned above, certain functional DGCs were transcribed at relatively low levels in bacteroids and exponential-phase cells, although a global active transcription of functional DGCs was observed at the stationary phase (reads per kilobase per million mapped reads [RPKM] > 67; Fig. 3). In line with these transcriptional profiles of functional DGCs, the average RPKM value of *mucR1* in stationary-phase cells was 12% and 52% of those in exponential-phase cells and bacteroids, respectively (Data Set S1). However, the relatively low transcription level of *mucR1* at the stationary phase can still be considered active transcription within the whole transcriptome (RPKM > 156; Data Set S1). qRT-PCR analyses of soybean nodules and the free-living cells in rich medium TY (optical density at 600 nm [OD_600_] of 1.2) showed that transcriptional levels of most functional DGCs were significantly upregulated in the **Δ***mucR1* mutant compared to the wild-type SF45436 (Fig. 6A and B), with more drastic changes in nodules. The transcription of functional PDE SFc33230 was also significantly upregulated in the **Δ***mucR1* mutant under both symbiotic and free-living conditions (Fig. 6A and B). Although these DGC/PDE genes have a scattered distribution pattern on the chromosome and chromid, the observed overall upregulation transcription profiles, particularly in nodules, imply that these genes are regulated by a shared regulation machinery.

10.1128/mBio.01192-21.6 DATA SET S1 RNA-seq analysis of SF45436. Download Data Set S1, XLSX file, 1.1 MB. Copyright © 2021 Li et al. 2021 Li et al. https://creativecommons.org/licenses/by/4.0/ This content is distributed under the terms of the Creative Commons Attribution 4.0 International license.

Cumulative evidence suggests the zinc-finger bearing MucR can be a global repressor preferring low-GC target sequences of low consensus (21, 39, 40). Sequence analysis revealed that the average %GC of upstream intergenic region for genes encoding functional or nonfunctional DGCs (Fig. 6C) is significantly lower than the genome average of 63.1% (*, *P < *0.05; **, *P < *0.01; *t* test) and the average value of the chromosome (63.45%, *P*  < 0.05; *t* test) and chromid (63.04%, *P*  < 0.05; *t* test) while indistinguishable from the average %GC of the symbiosis plasmid pSymA (59.92%, *P*  > 0.097; *t* test). Further electrophoretic mobility shift assay (EMSA) with the purified Sumo-MucR1 and its derivative MucR1 (with Sumo removed by HRV-3C protease) demonstrated that MucR1 can directly bind the promoter regions of genes encoding eight functional DGCs and the functional PDE SFc33230 (Fig. 6D) but not on three tested nonfunctional DGCs (Fig. S3). A clear gradient of band shift for test probes was observed when the ratio of MucR1 to probe was increased (Fig. 6D), suggesting oligomeric or multiple MucR1 binding events. This is in line with the hypothesis that MucR1 works in a similar nonspecific way in binding DNA as the well-known H-NS (histone-like nucleoid structuring protein) of E. coli (21, 40–43). The global repression of functional DGC genes located on different replicons by MucR1, particularly in nodules, represents a largely unexplored scenario in the evolution of the facultative life cycle of rhizobia.

10.1128/mBio.01192-21.3 FIG S3 Electrophoretic mobility shift assay (EMSA) with MucR1 and the promoters of three nonfunctional DGCs. The purified MucR1 with increasing concentrations (4.5 and 13.5 μM) were incubated with Cy5-labeled DNA probes. %GC of test probes are shown. Download FIG S3, PDF file, 0.1 MB. Copyright © 2021 Li et al. 2021 Li et al. https://creativecommons.org/licenses/by/4.0/ This content is distributed under the terms of the Creative Commons Attribution 4.0 International license.

The MucR/Ros family proteins are mainly found in alpha and deltaproteobacteria, particularly conserved in the former class that is enriched with various pathogenic and mutualistic bacteria associated with eukaryote hosts (21). Intensive studies of MucR/Ros homologs from *Agrobacterium*, *Sinorhizobium*, *Rhizobium*, *Mesorhizobium*, *Caulobacter*, and Brucella uncovered not only the conserved phenotype of rough colonies of the *mucR* or *ros* mutant but also their pleotropic transcriptional regulatory role in various cellular processes, such as the production of exopolysaccharides, motility and chemotaxis, cell cycle, ion uptake, and protein secretion systems (22, 38, 39, 44–53), some of which are involved in symbiosis and virulence. In the model rhizobium S. meliloti, for example, MucR directly activates the transcription of *exoY*, encoding a galactosyltransferase initiating the repeating unit assembly process during the biosynthesis of succinoglycan exopolysaccharide (54) that is essential for S. meliloti host invasion (48, 55). MucR can directly repress the transcription of Rem, which is a transcriptional activator of motility genes (56). It has been established that c-di-GMP is a ubiquitous second messenger modulating motility, biofilm formation, virulence, and cell cycle in bacteria (57), and several common cellular process regulated by both c-di-GMP and MucR recently have been reviewed for the free-living lifestyle of S. meliloti (58). This work further demonstrated that, at the nitrogen-fixing stage of mutualistic interaction between rhizobium and legumes, MucR can globally repress functional DGCs to downshift various energetically expensive processes induced by c-di-GMP. This strategic regulation mechanism also can be tested in other bacterium-host interactions.

### Conclusions.

**FIG 7:**
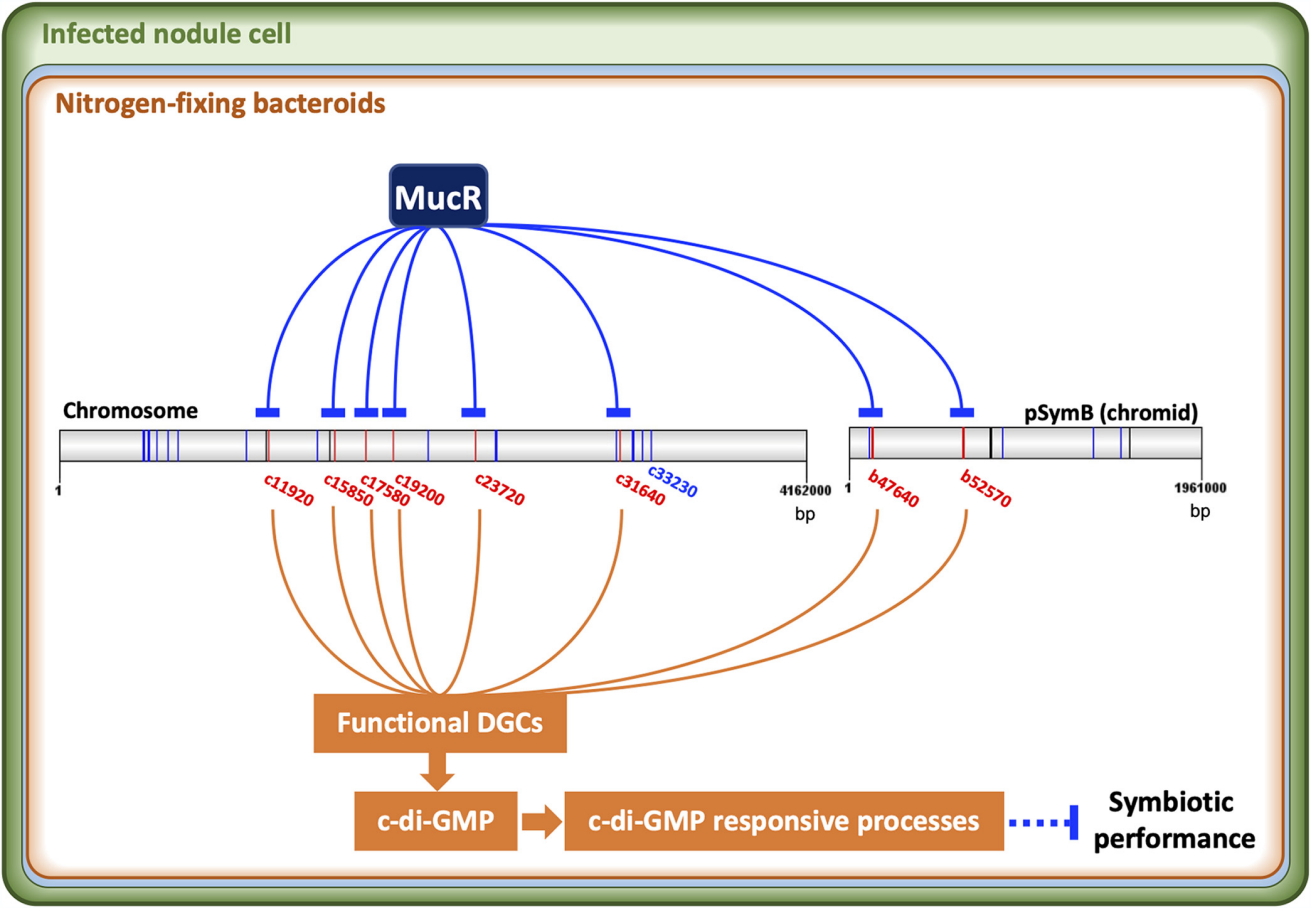
Global repression of functional DGC genes located on chromosome and chromid by MucR in efficient soybean nodules. Genes encoding functional and nonfunctional DGCs are in red and blue, respectively. Artificial accumulation of c-di-GMP in nodules by using PnifH-c17580 leads to upregulation of various c-di-GMP responsive processes and impairs symbiotic performance. Other regulators can be involved in the coordinated regulation of these DGC genes under different conditions but have not been identified yet. Circular replicons (chromosome and chromid) are linearized here for presentation purpose.

Rhizobia are characterized by their facultative symbiotic life cycle, in which various stimuli should be sensed and properly responded to. Dozens of c-di-GMP signaling components are present in rhizobia but are largely unexplored. This work made a systematic screening of functional DGCs in *S. fredii* SF45436 by using both *in vitro* and *in vivo* experiments (Fig. 1 and 2). The condition-dependent transcriptomic profiles suggest a general downregulation of functional DGC genes in soybean nodules (Fig. 3). An engineered nodule-specific accumulation of c-di-GMP led to an increased number of inefficient nodules, while no c-di-GMP could be detected in efficient nodules infected by the wild-type SF45436 and the PDE-overexpressing strain (Fig. 4). The elevated c-di-GMP induced various c-di-GMP-responsive processes, which are energetically costly and negatively regulated by the pleotropic regulator MucR1 in nodules (Fig. 5). It was further revealed that functional DGCs with scattered distributions in the multipartite genome of SF45436 were all directly repressed by MucR1 through oligomeric or multiple binding events on their promoter regions (Fig. 6). Collectively, these findings demonstrate a strategic repression of the c-di-GMP biosynthesis arsenal in legume nodules (Fig. 7), which represents a novel adaptation mechanism potentially explored by many other prokaryotes harboring a rich pool of c-di-GMP signaling components and their distinct global silencers, such as MucR (alphaproteobacteria, G-), H-NS and MvaT (gammaproteobacteria, G-), Lsr2 (actinobacteria, G^+^), and Rok (bacilli, G^+^) (21).

## MATERIALS AND METHODS

### Bacteria and culture conditions.

Bacterial strains and plasmids used in this work are shown in Table S1 in the supplemental material. E. coli was grown at 37°C in Luria broth (LB) medium. *S. fredii* SF45436 and its derivatives were grown at 28°C in TY medium (tryptone at 5 g/liter, yeast extract at 3 g/liter, and CaCl_2_ at 0.6 g/liter). The antibiotic concentrations used were 50 μg/ml kanamycin (Km), 10 μg/ml trimethoprim (Tmp), 30 μg/ml gentamicin (Gen), and 30 μg/ml carbenicillin (Cb).

10.1128/mBio.01192-21.4 TABLE S1 Strains and plasmids used in this study. Download Table S1, PDF file, 0.2 MB. Copyright © 2021 Li et al. 2021 Li et al. https://creativecommons.org/licenses/by/4.0/ This content is distributed under the terms of the Creative Commons Attribution 4.0 International license.

### Genetic procedures.

The primers used are listed in Table S2. All plasmid constructs in this work were verified by Sanger sequencing. Plasmids were transformed into E. coli DH5α (unless indicated) before conjugation into rhizobia with pRK2013 as the helper plasmid.

10.1128/mBio.01192-21.5 TABLE S2 Primers used in this work. Download Table S2, PDF file, 0.2 MB. Copyright © 2021 Li et al. 2021 Li et al. https://creativecommons.org/licenses/by/4.0/ This content is distributed under the terms of the Creative Commons Attribution 4.0 International license.

The Δ*mucR1* mutant was generated by removing the gentamicin resistance cassette within the Δ*mucR1*::*Gm* mutant constructed previously (22). The pCM157 plasmid carrying Cre recombinase that recognizes the *loxP* sites flanking the gentamicin cassette was introduced into the Δ*mucR1*::*Gm* mutant, and transconjugants sensitive to gentamicin were selected for subsequent screening of pCM157-cured strains (59). To construct in-frame deletion mutations of *SFc33230*, *SFc17580*, and *SFb52570*, a seamless assembly method was used. Briefly, gene-flanking regions of 500 to 1,000 bp were amplified using primers carrying 5′-homologous sequences containing the ends of SmaI restriction sites (CCC) in pJQ200SK (60). Two flanking fragments were mixed with SmaI-linearized pJQ200SK and incubated at 50°C for 15 min using a seamless cloning kit (Taihe Biotechnology), and positive transformants with correct sequences were used for conjugation with SF45436. Single-crossover clones resistant to Gen were selected for sequencing verification and subsequent cultivation in liquid TY medium for 36 h. The resulting liquid culture was subjected to double-crossover screening on a TY plate containing 7% (wt/vol) sucrose and Tmp as previously described (61).

Individual coding sequences for 26 DGCs (including YdeH from E. coli K-12), 20 GGDEF domains, and two EAL domains (SFc33230_EAL_ and SFb52570_EAL_) were amplified using primers harboring 5′-homologous sequences, including the ends of the NdeI restriction site (CAT) of pET28a(+), or primers carrying 5′-homologous sequences, including the ends of BamHI site (GGA) of pET30a-SUMO. These amplified fragments were individually subjected to seamless cloning with the plasmids pET28a(+) and pET30a-SUMO linearized by NdeI and BamHI, respectively. The resulting plasmids were then transformed into E. coli BL21(DE3) or Rosetta(DE3) before Congo red binding assay or protein purification experiments.

To construct pJQ-P_nifH_-c17580 and pJQ-P_nifH_-c33230_EAL_, a promoter region (496 bp of *SF45436_a46030*) and the upstream and downstream fragments of the coding sequences of *SFc17580* or *SF*c33230_EAL_ were amplified using primers containing the ends of the SmaI restriction site of pJQ200SK. These fragments were linked with pJQ200SK linearized by SmaI using the seamless assembly method described above.

The EZ-T Simple Zero pTOPO cloning kit (GenStar) was used to clone intergenic regions, which were then used as DNA probes in EMSA.

### Protein expression and purification.

E. coli BL21(DE3) or Rosetta(DE3) carrying the expression plasmids were grown in LB medium until the OD_600_ had reached 0.8. Gene expression was induced by adding 0.2 mM IPTG for 14 h at 18°C. The cells were harvested (4,000 × *g*, 5 min, 4°C) and resuspended in a lysis buffer (25 mM Tris-HCl, pH 8.0, 250 mM NaCl, 50 mM imidazole, 5% glycerol), supplemented with EDTA-free protease-inhibitor cocktail (1 tablet/50 ml buffer; Roche) and 200 μg/ml lysozyme. After sonication, the supernatant was loaded onto nickel columns washed with the same lysis buffer as described above and eluted with gradient imidazole elution from 100 mM to 500 mM. For the enzyme assay, the elution fractions were purified by size exclusion chromatography using a Superdex 200 10/30 column (GE Healthcare) and the SEC buffer (20 mM Tris-HCl, pH 8.0, 250 mM NaCl, 5% glycerol).

### Congo red binding assays.

E. coli BL21(DE3) or Rosetta(DE3) strains containing pET28a(+) or pET30a-SUMO were grown overnight, subcultured in fresh LB medium, and grown to an OD_600_ of 0.8. Next, 5 μl culture was dropped onto the LB agar medium containing Congo red (50 μg/ml) and IPTG (0.5 mM). Plates were incubated at 23°C, 28°C, and 37°C for 3 days before recording, and the Congo red binding phenotype at 23°C was more obvious under test conditions and was shown in Fig. 2 and Fig. S1.

### Western blotting.

To determine relative expression levels of cloned genes in E. coli strains used in Congo red binding assay, bacteria cultured in LB medium at an OD_600_ of 0.8 were subject to protein expression induced by 0.5 mM (final concentration) IPTG (23°C for 16 h). Cells from pre- and postinduction cultures (adjusted to OD_600_ of 0.8) were harvested by centrifugation (14,000 × *g* for 2 min, 4°C). The pellets were resuspended in SDS-loading buffer and lysed by boiling for 5 min. Each lysate was separated on SDS-PAGE gels and then transferred to nitrocellulose membrane. For immunodetection of individual proteins, HRP (horseradish peroxidase)-conjugated anti-His-Tag mouse monoclonal antibody (CWBIO, China) and ECL Western detection reagents (Solarbio, China) were used.

### Enzyme assays.

HPLC-MS was used to analyze DGC/PDE activity. The c-di-GMP synthesis assay was measured in a reaction mixture (100 μl) containing 75 mM Tris-HCl (pH 8.0), 250 mM NaCl, 25 mM KCl, 10 mM MgCl_2_·6H_2_O, 478 μM GTP (Sigma), and 1 μM protein (26). The c-di-GMP hydrolysis assay was measured in a reaction mixture (100 μl) consisting of 100 mM Tris-HCl (pH 8.0), 20 mM KCl, 25 mM MgCl_2_·6H_2_O, 340 μM c-di-GMP, and 1 μM protein. The mixtures were incubated at 37°C for 12 h and stopped by heating the sample for 5 min at 95°C (29). The supernatants obtained by centrifugation for 10 min at 12,500 × *g* were filtered through a 0.2-μm syringe filter. Nucleotides were separated and analyzed on a C_18_ reverse-phase column (62).

### Plant assays.

Symbiotic performance of *S. fredii* strains was tested on Glycine max cv. JD17. Seeds were surface sterilized, germinated, and inoculated as previously described (35). At 35 days postinoculation (dpi), leaf chlorophyll content for three leaflets of the third leaf was determined by a SPAD-502 m (Konica Minolta) and nodule numbers were counted. Three independent experiments were performed.

### Quantification of c-di-GMP in bacterial cultures and soybean nodules.

E. coli BL21(DE3) or Rosetta(DE3) cells carrying empty vectors or expression vectors were cultured and subject to IPTG induction as described above for Western blot analysis. Cells were harvested (4,000 × *g*, 8 min, 4°C) and washed by physiological saline twice. For nodule samples, nodules at 35 dpi were ground with mortar in liquid nitrogen. The resultant bacterial cells or nodule samples were resuspended with extraction solution (acetonitrile-methanol-water at 2:2:1 [vol/vol/vol]) as previously described (63). The resultant 600-μl extraction solution was analyzed by HPLC-MS/MS with c-di-GMP (MedChemExpress, USA) as an internal standard. To determine total protein concentrations of samples, all pellets were resuspended in 800 μl of 0.1 M NaOH and heated at 95°C for 30 min. The soluble samples were centrifuged at 12,000 × *g* for 5 min and the total protein content was then determined by using bicinchoninic acid (BCA) protein assay kit (ZOMANBIO). Three independent experiments were performed.

### RNA-seq analysis.

The bacterial cultures of SF45436 carrying pBBRMCS-3 grown in 50 ml TY medium were harvested during log phase (OD_600_, ∼0.5) or stationary phase (OD_600_, ∼4.2). RNA was isolated from bacterial cultures with the bacterial total RNA kit (ZOMANBIO). For bacteroid samples, nodules collected at 35 dpi were subject to RNA extraction using the Qiagen RNeasy minikit. Three biological replicates were performed. Strand-specific RNA sequencing was carried out by Novogene using an Illumina HiSeq platform (Illumina). Clean reads were mapped to the genome of SF45436 using Bowtie2 (default parameters) (23, 64). The number of unique mapped reads for each protein-coding gene was extracted from sorted bam files by HTseq-count (-a 0) (65). Reads per kilobase per million mapped reads (RPKM) were calculated for individual genes and are shown in Data Set S1.

### qRT-PCR.

SF45436 and its derivatives were grown in 50 ml of TY liquid medium overnight to an OD_600_ of 1.2. The RNA of bacteria was extracted as described above. Isolation of RNA from 35-dpi nodules of *G. max* cv. JD17 was performed using the total RNA kit (Promega). cDNA was synthesized by using the FastKing genomic DNA dispelling RT supermix (TIANGEN). qPCR was performed by using QuantStudioTM 6 Flex and 2× RealStar green mixture (Genstar). The 16S rRNA gene was used as the reference for normalization of gene expression. Three independent biological replicates were analyzed.

### EMSA.

The 45-bp Cy5-04750IR probe was generated by annealing the synthesized sense (5′-Cy5) and antisense single-stranded DNA. The other DNA probes were amplified by PCR with the pTOPO plasmid carrying intergenic regions as templates and labeled with Cy5 at 5′ ends, generating Cy5-DNA. The reaction mixture (10 μl) consisted of 0.5 mg/ml bovine serum albumin, 0.1 mg/ml sonicated salmon sperm DNA, 12.3 nM Cy5-DNA, 25 mM Tris-HCl (pH 8.0), 5% glycerol, 0.05% n-dodecyl-β-d-maltoside, and various concentrations of test proteins. The samples were incubated at 20°C for 30 min. Next, 1 μl HRV-3C protease (200 ng/μl; 10 mM dithiothreitol) was mixed well and incubated for a further 30 min. The samples were separated in a 6% TB polyacrylamide gel (no EDTA), and the gel was scanned with a Typhoon FLA 9000 imager (GE Healthcare).

### Bioinformatic and statistical analyses.

Protein domains were predicted using the NCBI Conserved Domains Database (https://www.ncbi.nlm.nih.gov/Structure/bwrpsb/bwrpsb.cgi). For predicting enzymatic activity of GGDEF, EAL, and PilZ domains, the retrieved protein sequences were aligned with those of proteins with demonstrated enzyme activity. For the GGDEF domain, it was predicted to be active in DGC function if the motif D_X(7 aa)_N_X(8 aa)_D_X(21 aa)_R_X_G_/S/A_GD_/E_EF was present. Domains containing the RxxD motif located five residues upstream of the GGDEF motif were considered to have an intact allosteric inhibitory site (I-site). For the EAL domain, E_X(55∼58 aa)_N_X(31 aa)_E_XX_E_(26 aa)_D_(20 aa)_K_(35 aa)_E was taken as evidence for putative PDE activity. For predicting c-di-GMP receptors carrying the PilZ domain, the motif R_XXX_R_(20∼30 aa)_D/N_X_S/A_xx_G was used (1). A domain was considered degenerate if at least one essential residue mentioned above did not match.

GraphPad Prism version 9.0.2 was used to perform statistical analysis, including *t* test and one-way and two-way ANOVA (analysis of variance), followed by multiple-comparison tests as shown in the figure legends (α = 0.05).

### Data availability.

Raw sequence data from our RNA-seq analyses can be accessed via NCBI Sequence Read Archive (PRJNA723738).
