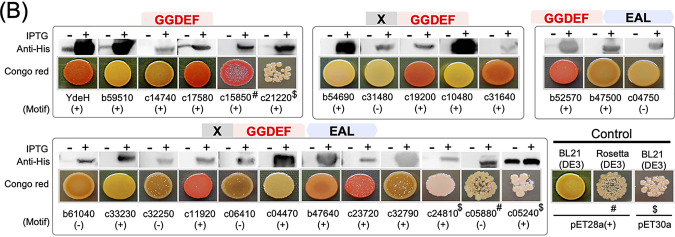# Correction for Li et al., “Global Transcriptional Repression of Diguanylate Cyclases by MucR1 Is Essential for *Sinorhizobium*-Soybean Symbiosis”

**DOI:** 10.1128/mbio.03369-21

**Published:** 2021-12-21

**Authors:** Meng-Lin Li, Jian Jiao, Biliang Zhang, Wen-Tao Shi, Wen-Hao Yu, Chang-Fu Tian

**Affiliations:** a State Key Laboratory of Agrobiotechnology, MOA Key Laboratory of Soil Microbiology, and Rhizobium Research Center, College of Biological Sciences, China Agricultural University, Beijing, China

## AUTHOR CORRECTION

Volume 12, issue 5, e01192-21, 2021, https://doi.org/10.1128/mBio.01192-21. In Fig. 2B of the original paper, the image of the Western blot that shows the expression level of c15850 using anti-His monoclonal antibody was duplicated in the panel for c21220. This duplication issue happened at the figure assembly stage and was not identified at submission and proof stages. The authors provide here Fig. 2B with the correct Western blot image in the c21220 panel, which does not change results and conclusions of the article. Nonetheless, the authors apologize to the readers for any inconvenience that this error may have caused.

**FIG 2:**