# Supplementary material for: Global Transcriptional Repression of Diguanylate Cyclases by MucR1 Is Essential for Sinorhizobium-Soybean Symbiosis
Source: mBio. 2021 Oct 26;12(5):e01192-21. doi: 10.1128/mBio.01192-21 (PMC8546604; doi:10.1128/mBio.01192-21)
Supplement: FIG S1 [file mbio.01192-21-sf001.pdf]

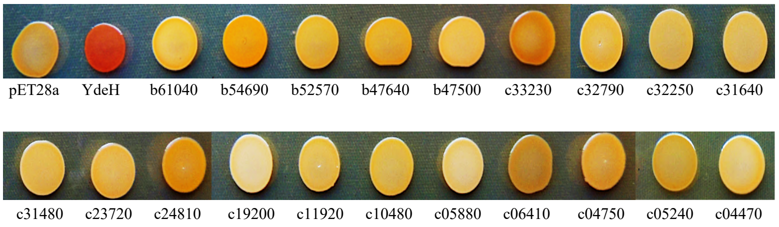

Fig S1. Congo red binding ability of *E. coli* strains harboring various GGDEF domains from corresponding proteins of SF45436. YdeH is a known functional DGC from *E. coli*.
