# Supplementary material for: Global Transcriptional Repression of Diguanylate Cyclases by MucR1 Is Essential for Sinorhizobium-Soybean Symbiosis
Source: mBio. 2021 Oct 26;12(5):e01192-21. doi: 10.1128/mBio.01192-21 (PMC8546604; doi:10.1128/mBio.01192-21)
Supplement: FIG S2 [file mbio.01192-21-sf002.pdf]

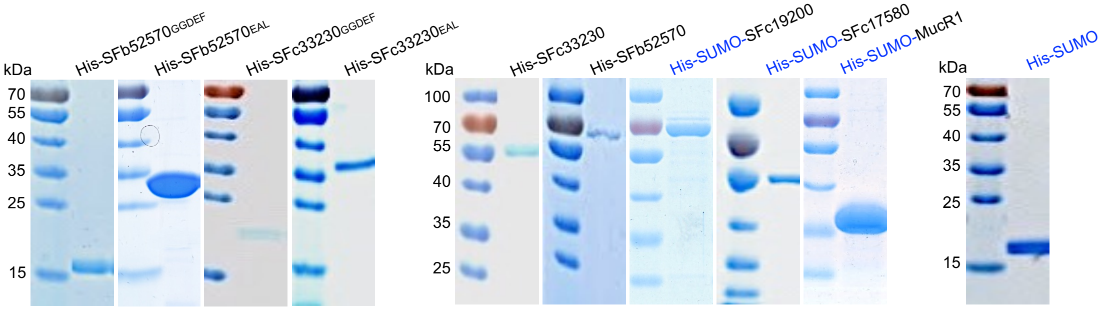

Fig S2. SDS-PAGE gels of purified proteins. His-SFc19200, His-SFc17580, and His-MucR1 were poorly soluble *in vitro*, and the His-SUMO versions were purified.
