# Supplementary material for: Global Transcriptional Repression of Diguanylate Cyclases by MucR1 Is Essential for Sinorhizobium-Soybean Symbiosis
Source: mBio. 2021 Oct 26;12(5):e01192-21. doi: 10.1128/mBio.01192-21 (PMC8546604; doi:10.1128/mBio.01192-21)
Supplement: FIG S3 [file mbio.01192-21-sf003.pdf]

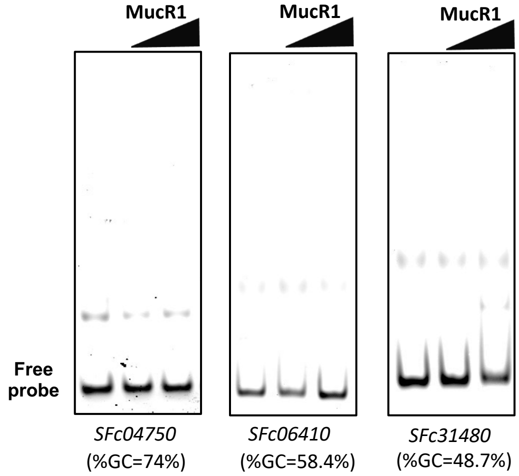

Fig S3. Electrophoretic mobility shift assay (EMSA) with MucR1 and the promoters of three nonfunctional DGCs. The purified MucR1 with increasing concentrations (4.5 and 13.5 μM) were incubated with Cy5-labeled DNA probes. %GC of test probes are shown.
