# Supplementary material for: Global Transcriptional Repression of Diguanylate Cyclases by MucR1 Is Essential for Sinorhizobium-Soybean Symbiosis
Source: mBio. 2021 Oct 26;12(5):e01192-21. doi: 10.1128/mBio.01192-21 (PMC8546604; doi:10.1128/mBio.01192-21)
Supplement: TABLE S1 [file mbio.01192-21-st001.pdf]

## Table S1. Strains and plasmids used in this study.

| Strains/Plasmids | Properties | Reference |
|---|---|---|
| ***Escherichia coli*** | | |
| DH5α | $F^-$Φ80*lacZ*Δ*M15*Δ(*lacZYA-argF*) U169*endA1recA1hsdR17* ($r_k^-$,$m_k^+$) *supE44* $\lambda^-$ *thi-1 gyrA96 relA1 phoA* | GenStar |
| BL21 (DE3) | $F^-$ *lon ompT hsdSB*(*rB*$^-$,*mB*$^-$)*dcm gal me131*(*DE3*) | GenStar |
| Rosetta (DE3) | *F- ompT hsdSB (rB-, mB-) gal dcm lacY1*(*DE3*) *pRARE* (*argU, argW, ilex, glyT, leuW, proL*), $Cam^r$ | Zomanbio |
| K12 | $F^-$, $\lambda^-$, *rph-1*[a] | This work |
| ***Sinorhizobium* strains** | | |
| SF45436 | *S. fredii* CCBAU45436 wild type, $Tmp^r$, $NA^r$ | (1) |
| Δ*c17580* | *S. fredii* CCBAU45436Δ*c17580*, $Tmp^r$, $NA^r$ | This work |
| Δ*b52570* | *S. fredii* CCBAU45436Δ*b52570*, $Tmp^r$, $NA^r$ | This work |
| Δ*c17580*Δ*b52570* | *S. fredii* CCBAU45436Δ*c17580*Δ*b52570*, $Tmp^r$, $NA^r$ | This work |
| Δ*c33230* | *S. fredii* CCBAU45436Δ*c33230*, $Tmp^r$, $NA^r$ | This work |
| Δ*c17580*.c | SF45436Δ*c17580* strain complemented with *SFc17580*, $Tmp^r$, $NA^r$ | This work |
| Δ*b52570*.c | SF45436Δ*b52570* strain complemented with *SFb52570*, $Tmp^r$, $NA^r$ | This work |
| $P_{nifH}$-DGC | *S. fredii* CCBAU45436 carrying DGC (*SFc17580*) driven by the *nifH* (*SFa46030*) promoter $P_{nifH}$, $Tmp^r$, $NA^r$ | This work |
| $P_{nifH}$-PDE | *S. fredii* CCBAU45436 carrying PDE (*SFc33230*$_{EAL}$) driven by the *nifH* (*SFa46030*) promoter $P_{nifH}$, $Tmp^r$, $NA^r$ | This work |
| Δ*mucR1-Gm* | *S. fredii* CCBAU45436Δ*mucR1::Gm*, $Tmp^r$, $Gen^r$ | (2) |
| Δ*mucR1* | *S. fredii* CCBAU45436Δ*mucR1*, $Tmp^r$, $NA^r$ | This work |
| SF83666 | *S. fredii* CCBAU83666 wild type, $Tmp^r$, $NA^r$ | (1) |
| SJ05684 | *S. sojae* CCBAU05684 wild type, $Tmp^r$, $NA^r$ | (1) |
| SS05631 | *S.* sp. CCBAU05631 wild type, $Tmp^r$, $NA^r$ | (1) |
| **Plasmids** | | |
| pET-28a (+) | Bacterial expression vector with T7 promoter, $Km^r$ | Novagen |
| pET-30a-SUMO | Bacterial expression vector with T7 promoter, $Km^r$ | Novagen |
| pCM157 | IncP plasmid providing expression of the Cre recombinase, $Tc^r$ | (3) |
| pJQ200SK | suicide plasmid with *sacB* selectable marker, $Gm^r$ | (4) |
| pRK2013 | ColE1, $Tra^+$, $Km^r$ | (5) |
| pTOPO-T | Cloning vector, $Amp^r$, $Cb^r$ | GenStar |
| pBBRMCS-3 | Broad-host-range vector, $Tc^r$ | (6) |
| **Plasmids for gene overexpression** | | |
| pEydeH | pET28a (+) overexpressing *ydeH*, $Km^r$ | This work |
| pET-SFb59510 | pET28a (+) overexpressing *SFb59510*, $Km^r$ | This work |
| pET-SFc21220 | pET28a (+) overexpressing *SFc21220*, $Km^r$ | This work |
| pET-SFc17580 | pET28a (+) overexpressing *SFc17580*, $Km^r$ | This work |
| pET-SFc15850 | pET28a (+) overexpressing *SFc15850*, $Km^r$ | This work |
| pET-SFb54690 | pET28a (+) overexpressing *SFb54690*, $Km^r$ | This work |
| pET-SFc31480 | pET28a (+) overexpressing *SFc31480*, $Km^r$ | This work |
| pET-SFc19200 | pET28a (+) overexpressing *SFc19200*, $Km^r$ | This work |
| pET-SFc10480 | pET28a (+) overexpressing *SFc10480*, $Km^r$ | This work |

| Strains/Plasmids | Properties | Reference |
|---|---|---|
| pET-SFc31640 | pET28a (+) overexpressing *SFc31640*, $Km^r$ | This work |
| pET-SFb52570 | pET28a (+) overexpressing *SFb52570*, $Km^r$ | This work |
| pET-SFb47500 | pET28a (+) overexpressing *SFb47500*, $Km^r$ | This work |
| pET-SFc04750 | pET28a (+) overexpressing *SFc04750*, $Km^r$ | This work |
| pET-SFb61040 | pET28a (+) overexpressing *SFb61040*, $Km^r$ | This work |
| pET-SFc33230 | pET28a (+) overexpressing *SFc33230*, $Km^r$ | This work |
| pET-SFc32250 | pET28a (+) overexpressing *SFc32250*, $Km^r$ | This work |
| pET-SFc11920 | pET28a (+) overexpressing *SFc11920*, $Km^r$ | This work |
| pET-SFc06410 | pET28a (+) overexpressing *SFc06410*, $Km^r$ | This work |
| pET-SFc05240 | pET28a (+) overexpressing *SFc05240*, $Km^r$ | This work |
| pET-SFb47640 | pET28a (+) overexpressing *SFb47640*, $Km^r$ | This work |
| pET-SFc23720 | pET28a (+) overexpressing *SFc23720*, $Km^r$ | This work |
| pET-SFc32790 | pET28a (+) overexpressing *SFc32790*, $Km^r$ | This work |
| pET-SFc24810 | pET28a (+) overexpressing *SFc24810*, $Km^r$ | This work |
| pET-SFc05880 | pET28a (+) overexpressing *SFc05880*, $Km^r$ | This work |
| pET-SFc04470 | pET28a (+) overexpressing *SFc04470*, $Km^r$ | This work |
| pET-$SFb61040_{GGDEF}$ | pET28a (+) overexpressing $SFb61040_{GGDEF}$ | This work |
| pET-$SFb54690_{GGDEF}$ | pET28a (+) overexpressing $SFb54690_{GGDEF}$ | This work |
| pET-$SFb52570_{GGDEF}$ | pET28a (+) overexpressing $SFb52570_{GGDEF}$ | This work |
| pET-$SFb47640_{GGDEF}$ | pET28a (+) overexpressing $SFb47640_{GGDEF}$ | This work |
| pET-$SFb47500_{GGDEF}$ | pET28a (+) overexpressing $SFb47500_{GGDEF}$ | This work |
| pET-$SFc33230_{GGDEF}$ | pET28a (+) overexpressing $SFc33230_{GGDEF}$ | This work |
| pET-$SFc32790_{GGDEF}$ | pET28a (+) overexpressing $SFc32790_{GGDEF}$ | This work |
| pET-$SFc32250_{GGDEF}$ | pET28a (+) overexpressing $SFc32250_{GGDEF}$ | This work |
| pET-$SFc31640_{GGDEF}$ | pET28a (+) overexpressing $SFc31640_{GGDEF}$ | This work |
| pET-$SFc31480_{GGDEF}$ | pET28a (+) overexpressing $SFc31480_{GGDEF}$ | This work |
| pET-$SFc23720_{GGDEF}$ | pET28a (+) overexpressing $SFc23720_{GGDEF}$ | This work |
| pET-$SFc24810_{GGDEF}$ | pET28a (+) overexpressing $SFc24810_{GGDEF}$ | This work |
| pET-$SFc19200_{GGDEF}$ | pET28a (+) overexpressing $SFc19200_{GGDEF}$ | This work |
| pET-$SFc11920_{GGDEF}$ | pET28a (+) overexpressing $SFc11920_{GGDEF}$ | This work |
| pET-$SFc10480_{GGDEF}$ | pET28a (+) overexpressing $SFc10480_{GGDEF}$ | This work |
| pET-$SFc05880_{GGDEF}$ | pET28a (+) overexpressing $SFc05880_{GGDEF}$ | This work |
| pET-$SFc06410_{GGDEF}$ | pET28a (+) overexpressing $SFc06410_{GGDEF}$ | This work |
| pET-$SFc04750_{GGDEF}$ | pET28a (+) overexpressing $SFc04750_{GGDEF}$ | This work |
| pET-$SFc05240_{GGDEF}$ | pET28a (+) overexpressing $SFc05240_{GGDEF}$ | This work |
| pET-$SFc04470_{GGDEF}$ | pET28a (+) overexpressing $SFc04470_{GGDEF}$ | This work |
| pSumo-SFc17580 | pET30a-SUMO overexpressing *SFc17580* | This work |
| pSumo-SFc19200 | pET30a-SUMO overexpressing *SFc19200* | This work |
| pSumo-SFc19200 | pET30a-SUMO overexpressing *SFc19200* | This work |
| pSumo-SFc21220 | pET30a-SUMO overexpressing *SFc21220* | This work |
| pSumo-SFc24810 | pET30a-SUMO overexpressing *SFc24810* | This work |
| pSumo-SFc05240 | pET30a-SUMO overexpressing *SFc05240* | This work |
| pET-$SFc33230_{EAL}$ | pET28a (+) overexpressing EAL domain of *SFc33230* | This work |

| Strains/Plasmids | Properties | Reference |
|---|---|---|
| pET-SFb52570$_{EAL}$ | pET28a (+) overexpressing EAL domain of *SF52570* | This work |
| pSumo-MucR1 | pET30a-SUMO overexpressing *SFc06950* | (7) |
| **Plasmids for deletion and complementation** | | |
| pJQ200SK-*c33230* | pJQ200SK carrying internal fragment of SF*c33230*, Gm$^r$ | This work |
| pJQ200SK-*c17580* | pJQ200SK carrying internal fragment of SF*c17580*, Gm$^r$ | This work |
| pJQ200SK-*b52570* | pJQ200SK carrying internal fragment of SF*b52570*, Gm$^r$ | This work |
| pJQ200SK-*c17580*.c | pJQ200SK carrying SF*c17580* for *in situ* complementation, Gm$^r$ | This work |
| pJQ200SK-*b52570*.c | pJQ200SK carrying SF*b52570* for *in situ* complementation, Gm$^r$ | This work |
| **Promoter fusion plasmids** | | |
| pJQ-P$_{nifH}$-c17580 | pJQ200SK carrying the upstream and downstream fragments of intergenic region of *SFc17570* and *SFc17580*, Gm$^r$ | This work |
| pJQ-P$_{nifH}$-c33230$_{EAL}$ | pJQ200SK carrying the upstream and downstream fragments of intergenic region of *SFc33220* and *SFc33230*, Gm$^r$ | This work |
| **Plasmids for amplification of intergenic region for EMSA** | | |
| pTO-c17580 | pTOPO-T carrying intergenic region of *SFc17570* and *SFc17580*, Cb$^r$ | |
| pTO-c19200 | pTOPO-T carrying intergenic region of *SFc19200* and *SFc19210*, Cb$^r$ | |
| pTO-c23720 | pTOPO-T carrying intergenic region of *SFc23710* and *SFc23720*, Cb$^r$ | |
| pTO-c31640 | pTOPO-T carrying intergenic region of *SFc31640* and *SFc31650*, Cb$^r$ | |
| pTO-c33230 | pTOPO-T carrying intergenic region of *SFc33220* and *SFc33230*, Cb$^r$ | |
| pTO-b47640 | pTOPO-T carrying intergenic region of *SFb47640* and *SFb47650*, Cb$^r$ | |
| pTO-b52570 | pTOPO-T carrying intergenic region of *SFb52570* and *SFb52580*, Cb$^r$ | |
| pTO-c15850 | pTOPO-T carrying intergenic region of *SFc15840* and *SFc15850*, Cb$^r$ | |
| pTO-c11920 | pTOPO-T carrying intergenic region of *SFc11910* and *SFc11920*, Cb$^r$ | |
| pTO-c06410 | pTOPO-T carrying intergenic region of *SFc06400* and *SFc06410*, Cb$^r$ | |
| pTO-c31480 | pTOPO-T carrying intergenic region of *SFc31480* and *SFc31490*, Cb$^r$ | |

## References


1. Tian CF, et al. (2012) Comparative genomics of rhizobia nodulating soybean suggests extensive recruitment of lineage-specific genes in adaptations. *Proc Natl Acad Sci U S A* 109(22):8629–8634.
2. Jiao J, et al. (2016) MucR is required for transcriptional activation of conserved ion transporters to support nitrogen fixation of *Sinorhizobium fredii* in soybean nodules. *Mol Plant-Microbe Interact* 29(5):352–361.
3. Marx CJ, Lidstrom ME (2002) Broad-host-range *cre-lox* system for antibiotic marker recycling in Gram-negative bacteria. *Biotechniques* 33(5):1062–1067.
4. Quandt J, Hynes MF (1993) Versatile suicide vectors which allow direct selection for gene replacement in Gram-negative bacteria. *Gene* 127(1):15–21.
5. Khan SR, Gaines J, Roop RM, Farrand SK (2008) Broad-host-range expression vectors with tightly regulated promoters and their use to examine the influence of TraR and TraM expression on Ti plasmid quorum sensing. *Appl Environ Microbiol* 74(16):5053–62.
6. Kovach ME, et al. (1995) Four new derivatives of the broad-host-range cloning vector

pBBR1MCS, carrying different antibiotic-resistance cassettes. *Gene* 166(1):175–176.

7. Jiao J, et al. (2021) The zinc-finger bearing xenogeneic silencer MucR in α-proteobacteria balances adaptation and regulatory integrity. ISME J (Accepted).
