# Supplementary material for: Global Transcriptional Repression of Diguanylate Cyclases by MucR1 Is Essential for Sinorhizobium-Soybean Symbiosis
Source: mBio. 2021 Oct 26;12(5):e01192-21. doi: 10.1128/mBio.01192-21 (PMC8546604; doi:10.1128/mBio.01192-21)
Supplement: TABLE S2 [file mbio.01192-21-st002.pdf]

Table S2. Primers used in this work.

Name	Sequences (5'-3’)	Purpose
ydeH-NdeI-F	GTGCCGCGCGGCAGCCAT**ATG**ATCAAGAAGACAACGG	For construction of pET28a
ydeH-NdeI-R	ACCAGTCATGCTAGCCATTTAAACTCGGTTAATCACATTTTGTTCG	(+) carrying *ydeH*
Gb61040-NdeI-F	TGCCGCGCGGCAGCCATAATATCGACAGCCTCACGCACCTGCCG	
Gb61040-NdeI-R	TCCACCAGTCATGCTAGCCATTCACACGGTCCGCCCGGTCTTGTTCTGCT	
Gb52570-NdeI-F	CCTGGTGCCGCGCGGCAGCCATCAGGCAACCCATGACGTGTTGAC	
Gb52570-NdeI-R	TGTCCACCAGTCATGCTAGCCATTCACGTGAATATCCTTGCCCGGCC	
Gb54690-NdeI-F	GTGCCGCGCGGCAGCCATTACACCGACGAACTGACCGGGGTCGCCAA	
Gb54690-NdeI-R	CCACCAGTCATGCTAGCCATTCAGTGAATTTGGTCCCGGCCGGCGAGCTTG	
Gb47640-NdeI-F	GTGCCGCGCGGCAGCCATACCGATCACCTGACAGGGCTCCTCAA	
Gb47640-NdeI-R	TCCACCAGTCATGCTAGCCATTCAGTTCTTGCCGTTGTGCTTGCGGGCAT	
Gb47500-NdeI-F	GTGCCGCGCGGCAGCCATCACACCGACGCCCTGACCGGTCTCAACAA	
Gb47500-NdeI-R	TCCACCAGTCATGCTAGCCATTCACACGGTGGTGTTGCGGCCGCCCT	
Gc33230-NdeI-F	GTGCCGCGCGGCAGCCATAATACCGACTCGCTGACGGGCCTCGACAAC	
Gc33230-NdeI-R	TGTCCACCAGTCATGCTATCAGCCATTCACCGGGCGCAGTTCCGGCC	
Gc32790-NdeI-F	GTGCCGCGCGGCAGCCATCTGCACGACCCCTTGACTGGCCTGCCGAA	
Gc32790-NdeI-R	CACCAGTCATGCTAGCCATTCACCTGTTGCGGCCGCGCCTCTTGGCCTCA	
Gc32250-NdeI-F	GTGCCGCGCGGCAGCCATCGTTTCGATACGCTGACCGGACTGCCGAGC	
Gc32250-NdeI-R	TCCACCAGTCATGCTAGCCATTCAACACCCTCCCCTGTCGCGCGATTTCG	
Gc31640-NdeI-F	GTGCCGCGCGGCAGCCATACGGATGGATTGACCGGCATCGTCAACC	
Gc31640-NdeI-R	TCCACCAGTCATGCTAGCCATTCAGATGCGGTTGCGGCCGCGCATCTT	
Gc31480-NdeI-F	GTGCCGCGCGGCAGCCATGAACTCACCCATGTCCCCGACCGGCGCCATA	For construction of pET28a
Gc31480-NdeI-R	TCCACCAGTCATGCTAGCCATTCACACCCGGTCCCGGCCCTCGCGCTT	(+) carrying sequences
Gc24810-NdeI-F	GTGCCGCGCGGCAGCCATCACCACGATGCACTGACCGATCTTTCCAACC	coding for GGDEF domains
Gc24810-NdeI-R	TCCACCAGTCATGCTAGCCATTCATCCCTTGCCCGTCTGCTTGGCACGGT	
Gc23720-NdeI-F	GTGCCGCGCGGCAGCCATACCGATCACCTGACCGGGCTCCTCAA	
Gc23720-NdeI-R	TCCACCAGTCATGCTAGCCATTCACTGCAGGCCATTCTTGCCGTCGCG	
Gc19200-NdeI-F	GTGCCGCGCGGCAGCCATGACTACCTGACGGGAATCTTCAACCGCCG	
Gc19200-NdeI-R	TCCACCAGTCATGCTAGCCATTCAGATGACACGGTTTCGTCCGGCATGTTTG	
Gc11920-NdeI-F	GTGCCGCGCGGCAGCCATAACCACGACAGCCTGAGCGGATTGCTC	
Gc11920-NdeI-R	TCCACCAGTCATGCTAGCCATTCACACCGCCTGGCCCTTGCCGTTTTCCT	
Gc06410-NdeI-F	GTGCCGCGCGGCAGCCATCCGTTGACCGGCCTGCCAGGCAATC	
Gc06410-NdeI-R	TCCACCAGTCATGCTAGCCATTCACGAGCGAAAATCATCGATGAGCCGCATC	
Gc05880-NdeI-F	GTGCCGCGCGGCAGCCATGACAACCTCACCGGCCTGCCCAACCGAC	
Gc05880-NdeI-R	TCCACCAGTCATGCTAGCCATTCAGACGCGGTTGCCGCCCTCCTTCTTGG	
Gc05240-NdeI-F	GTGCCGCGCGGCAGCCATACCGACACGCTCACCGGCATCAACAATG	
Gc05240-NdeI-R	TCCACCAGTCATGCTAGCCATTCACTGGCGGAGCCGGGTACGCTTGTCG	
Gc04750-NdeI-F	GTGCCGCGCGGCAGCCATGATCCGTTGACGGGCCTGGGCAATGCAC	
Gc04750-NdeI-R	TCCACCAGTCATGCTAGCCATTCAGATCTGGCCGCGGCCGCGGCGTTTCG	
Gc04470-NdeI-F	GTGCCGCGCGGCAGCCATCGTCATGACAGCCTGACCGGGCTGCCCAA	
Gc04470-NdeI-R	TCCACCAGTCATGCTAGCCATTCACGCCGCCTTTGCCCGATACATGGCGAGG	
Gc10480-NdeI-F	GTGCCGCGCGGCAGCCATACCGACGACCTCACCGGCCTTCACAACCGC	
Gc10480-NdeI-R	TCCACCAGTCATGCTAGCCATTCAGACGACCCGGTTGCGCCCGTTGCTCTTG	

Name	Sequences (5'-3')	Purpose
b59510-NdeI-F	GTGCCGCGCGGCAGCCAT**ATG**CAGCTTCAGG	
b59510-NdeI-R	TCCACCAGTCATGCTAGCCATTCACTGACGAATCCGATCTGG	
c17580-NdeI-F	GTGCCGCGCGGCAGCCAT**ATG**CAGACGGCAACT	
c17580-NdeI-R	TCCACCAGTCATGCTAGCCATTTATCGGCGATAGATCAGC	
c21220-NdeI-F	GTGCCGCGCGGCAGCCAT**ATG**AGCATTTTCCCCGCC	
c21220-NdeI-R	TCCACCAGTCATGCTAGCCATTCAGGCGGTGTGGCGCAACCG	
c15850-NdeI-F	GTGCCGCGCGGCAGCCAT**ATG**GGTGGTGCGATTTCCC	
c15850-NdeI-R	TCCACCAGTCATGCTAGCCATTCAAGACCGCCCCACCCGCCGCG	
c14740-NdeI-F	GTGCCGCGCGGCAGCCAT**ATG**CCATACGCCAATCTCATG	
c14740-NdeI-R	TCCACCAGTCATGCTAGCCATTCAGCGCCCGACGACGCAGTTGC	
b54690-NdeI-F	GTGCCGCGCGGCAGCCAT**ATG**GCGAAAGATATCAGGCGACTCTTTGAAC	
b54690-NdeI-R	TCCACCAGTCATGCTAGCCATTTATCCCGCGACTGCAGATCGGTCTGGAT	
c31480-NdeI-F	GTGCCGCGCGGCAGCCAT**ATG**ACCGGTGATTACGTCAGG	
c31480-NdeI-R	TCCACCAGTCATGCTAGCCATCTACGAGGTCTGATCCGGGC	
c19200-NdeI-F	GTGCCGCGCGGCAGCCAT**GTG**AGATTGCGCCGCGCGTCGGGACG	
c19200-NdeI-R	TCCACCAGTCATGCTAGCCATTTAGGATGCGAGCGCATCGAGCAGCGCAAGC	
c10480-NdeI-F	GTGCCGCGCGGCAGCCAT**ATG**ACTGCGCGCATCCTCGTTGTCGATGACG	
c10480-NdeI-R	TCCACCAGTCATGCTAGCCATTTACGCGGCCGCCGCGACGACCCGGTT	
c31640-NdeI-F	GTGCCGCGCGGCAGCCAT**ATG**AATGGCATCTGGGGGCAGTTTGTTGGC	
c31640-NdeI-R	TCCACCAGTCATGCTAGCCATCTAACCGGCCATTTTGACGTCCGCCGC	
b52570-NdeI-F	CCTGGTGCCGCGCGGCAGCCAT**ATG**CCTGACACGGATGCGAC	For construction of pET28a (+) carrying sequences coding for DGCs
b52570-NdeI-R	TGTCCACCAGTCATGCTAGCCATTCAGGTCCTGAGAGCCCCC	
b47500-NdeI-F	CCTGGTGCCGCGCGGCAGCCAT**ATG**ATTGGAAAACCGCTGAGCAAGC	
b47500-NdeI-R	TGTCCACCAGTCATGCTAGCCATTTATCGCAGG GCGAGC	
c04750-NdeI-F	CCTGGTGCCGCGCGGCAGCCAT**ATG**ATGCTCG CCGAGCTTGC	
c04750-NdeI-R	TGTCCACCAGTCATGCTAGCCATTCATTCCCGAGCGATCAATTCCAGCACCAACCC	
c33230-NdeI-F	CCTGGTGCCGCGCGGCAGCCAT**ATG**AAGCACACGCTGGCCGACGGAACATGATCT	
c33230-NdeI-R	TGTCCACCAGTCATGCTAGCCATTCAGCGCGGCTTTGCCCGAG	
c32250-NdeI-F	TGGTGCCGCGCGGCAGCCAT**ATG**ACGCGCA ATGAACAGGG C	
c32250-NdeI-R	GTCCACCAGTCATGCTAGCCATCTAGGCGATGTTCGAGCGGGAG	
c11920-NdeI-F	CCTGGTGCCGCGCGGCAGCCAT**ATG**TCGTCTAATTCCGCCGACCGG	
c11920-NdeI-R	TGTCCACCAGTCATGCTAGCCATTCAAGCCGCCGAAGCCTCATGAAG	
c06410-NdeI-F	CCTGGTGCCGCGCGGCAGCCAT**ATG**CCCGCCG CCCCCGTAG	
c06410-NdeI-R	TGTCCACCAGTCATGCTAGCCATTCATTCCCGAGCGATCAATTCCAGCACCAACCC	
c05240-NdeI-F	TGCCGCGCGGCAGCCAT**GTG**ACACTCA GCAAGCGCGC	
c05240-NdeI-R	CACCAGTCATGCTAGCCATTCACCGGTGCGCTTCCC	
b47640-NdeI-F	GTGCCGCGCGGCAGCCAT**ATG**AATACGCATTCGGTCGAGAGCCGC	
b47640-NdeI-R	TCCACCAGTCATGCTAGCCATTTAATAGGTTTCGATCTTCAGCTTTTCCTGAGCC	
c23720-NdeI-F	CCTGGTGCCGCGCGGCAGCCAT**ATG**ATGCACTCGGTGGAAAG CCG	
c23720-NdeI-R	TGTCCACCAGTCATGCTAGCCATCTAGGCAGGAGTGCTGGCCTTC	
c32790-NdeI-F	GTGCCGCGCGGCAGCCAT**GTG**CCAAAAATCAAAGGTCAATATTTTGGGAAACACCGG CTC	
c32790-NdeI-R	TGTCCACCAGTCATGCTAGCCATTCAGGCGGCGCGCCACTTTC	

Name	Sequences (5'-3')	Purpose
c24810-NdeI-F	CCTGGTGCCGCGCGGCAGCCAT**ATG**TTCTCAGTAATTTCGTGCATAAGCGATCGC	
c24810-NdeI-R	TGTCCACCAGTCATGCTAGCCATCTACGCCTCGGCGTGTTGAAG	
c05880-NdeI-F	CCTGGTGCCGCGCGGCAGCCAT**ATG**CCCCTGA CCCGCAAGC	
c05880-NdeI-R	TGTCCACCAGTCATGCTAGCCATTTACGCCCTCTTCATGAGCGGAAATCG	
c04470-NdeI-F	CCTGGTGCCGCGCGGCAGCCAT**ATG**TTCAAGATTCTCTCGTGCCTCGTCGTG	
c04470-NdeI-R	TGTCCACCAGTCATGCTAGCCATTCAGGCGGCCTCGGTCCGGAC	
b61040-NdeI-F	CCTGGTGCCGCGCGGCAGCCAT**ATG**GGCGAGTGGGGGCGAAC	
b61040-NdeI-R	TGTCCACCAGTCATGCTAGCCATTCAGGCAGAAACCTTGCCGGGC	
mucR1-BamHI-F	GTTCCAGGGGCCCCTGGGA**ATG**GGTCATCACCATCATCATCACGGG	For construction of pET30a-SUMO carrying individual genes
mucR1-BamHI-R	TGGTGCTCGAGCGGCGGGGATTACTTGCCGCGACGCTTGCG	
c17580-BamHI-F	GTTCCAGGGGCCCCTGGGA**ATG**CAGACGGCAACTTC	
c17580-BamHI-R	TGGTGCTCGAGCGGCGGGGATTATCGGCGATAGATCAGCC	
c19200-BamHI-F	GTTCCAGGGGCCCCTGGGATCC**GTG**AGATTGCGCCGCGCGTC	
c19200-BamHI-R	TGGTGCTCGAGCGGCGGGGATCCTTAGGATGCGAGCGCATCGAGCAG	
c21220-BamHI-F	GTTCCAGGGGCCCCTGGGA**ATG**AGCATTTTCCCCGCC	
c21220-BamHI-R	TGGTGCTCGAGCGGCGGGGATCAGGCGGTGTGGCGCAACCG	
c24810-BamHI-F	GTTCCAGGGGCCCCTGGGATCC**ATG**TTCTCAGTAATTTCGTGCATAAGCGATCG	
c24810-BamHI-R	TG**GTGCTC**GAGCGGCGGGGATCCCTACGCCTCGGCGTGTTGAAG	
c05240-BamHI-F	CAGGGGCCCCTGGGATCC**GTG**ACACTCAGCAAGCGCG	
c05240-BamHI-R	CTCGAGCGGCGGGGATCCTCACCGGTGCGCTTCCC	
Ec33230-NdeI-F	CCTGGTGCCGCGCGGCAGCCATAACATCGCCGCCCTGTTGAAAATCGC CATC	For construction of pET28a (+) carrying sequences coding for EAL domains
Ec33230-NdeI-R	TGTCCACCAGTCATGCTAGCCATTCACGCTGGCCGGATGTTCGGCG	
Eb52570-NdeI-F	CCTGGTGCCGCGCGGCAGCCATATGCGCAGCCTGCGCTCAGCCCTTCAGGAGGGAC	
Eb52570-NdeI-R	TGTCCACCAGTCATGCTAGCCATTCACGCGGCTGCCGGGAGCGGG	
c33230-SmaI-UF	ATCGAATTCCTGCAGCCCTGGATGATCACCGGCGCATC	For *SFc33230* deletion
c33230-UR	GATCTCAATTGCGTCGGCCAGC	
c33230-DF	GACGCAATTGAGATCAGACGTTCCGGCTGTCCGCG	
c33230-SmaI-DR	AGAACTAGTGGATCCCCCGTGGTCGCCAACGGGCAGTTG	
c17580-SmaI-UF	CTTGATATCGAATTCCTGCAGCCCTTCAACCCGCTGATGGCCTAC	For *SFc17580* deletion
c17580-UR	GAAGTTGCCGTCTGCATATGGC	
c17580-DF	TATGCAGACGGCAACTTCCGCCGATAACAAGGTCAAGCTTTGC	
c17580-SmaI-DR	GCTCTAGAACTAGTGGATCCCCCGACGATCTGGCGGTTGTCCG	
b52570-SmaI-UF	ATCGAATTCCTGCAGCCCAATTGATCGCCAGGTCCAGGAG	For *SFb52570* deletion
b52570-UR	ATTTCCTGGGCTTCCCGCT	
b52570-DF	GGAAGCCCAGGAAATCTCTCGTCGTAAAGGGAGACGAGC	
b52570-SmaI-DR	AGAACTAGTGGATCCCCCTTGGCGATCTCGCCATCGATC	
c17580.c-SmaI-F	CTTGATATCGAATTCCTGCAGCCCTTCAACCCGCTGATGGCCTAC	For complementation of SF45436Δ*c17580*
c17580.c-SmaI-R	GCTCTAGAACTAGTGGATCCCCCGACGATCTGGCGGTTGTCCG	
b525700.c-SmaI-F	ATCGAATTCCTGCAGCCCAATTGATCGCCAGGTCCAGGAG	For complementation of SF45436Δ*b52570*
b52570.c-SmaI-R	AGAACTAGTGGATCCCCCTTGGCGATCTCGCCATCGATC	
pJQc17580-SmaI-UF	GATATCGAATTCCTGCAGCCCAGTTCTCCGGTTATGCGGCG	For construction of pJQ-P_{nifH}-c17580
pJQc17580-UR	TCAGGCAGGGCCGATCATC	
P_{a46030}-DGC-F	CGATGATCGGCCCTGCCTGA TGGGGACCACACCGTCAGC	

Name	Sequences (5'-3’)	Purpose
P_{a46030}-DGC-R	GTTGCTTTCCTTCGTTGTTCGAAACCATCGC	
pJQc17580-DF	GAACAACGAAGGAAAGCAACATGCAGACGGCAACTTCGAAC	
pJQc17580-SmaI DR	TCTAGAACTAGTGGATCCCCCTTATCGGCGATAGATCAGCCAGTTC	
pJQc33230$_{EAL}$-SmaI -UR	TGCAGCATTGTTCGGCACATTAG	
P_{a46030}-PDE-F	ACTAATGTGCCGAACAATGCTGCA TGGGGACCACACCGTCAGC	For construction of pJQ-P_{nifH}-c33230$_{EAL}$
P_{a46030}-PDE-R	GTTGCTTTCCTTCGTTGTTCGAAACCATCGC	
pJQc33230$_{EAL}$-DF	GAACAACGAAGGAAAGCAAC ATGAACATCGCCGCCCTGTTGAAAATCG	
pJQc33230$_{EAL}$-SmaI -DR	TCTAGAACTAGTGGATCCCCCTGATGCATGCCGCCCAAAAGTGC	
M13F	TGTAAAACGACGGCCAGT	Standard sequencing primers for pJQ200SK
M13R	CAGGAAACAGCTATGACC	
T7	TAATACGACTCACTATAGGG	Standard sequencing primers for pET28a (+) and pET30a-SUMO
T7 ter	TGCTAGTTATTGCTCAGCGG	
mucR1-interF	ACCTTGGTGACGGGTTTCGC	Verification primers for *mucR1* deletion
mucR1-interR	GCCGGACTGAGACAGCC	
c33230-interF	GCTGTCCTTCTCTCCGGCG	
c33230-interR	CACCTTCCTGGTGCCCGTAG	Verification primers for *c33230* deletion
c33230-outerF	TGCCAGACGCTCTGGTCGAAC	
c33230-outerR	CGACGATCGGTTCCCGCAATAC	
c17580-interF	ACTACGAGGCCTATCTGGGCTC	
c17580-interR	GGTCGCTTCGCTCAGAATGTCG	Verification primers for *c17580* deletion
c17580-outerF	TCACGCGTCGCTCACCTTC	
c17580-outerR	GCAGCATTGGCGACCGTTG	
b52570-interF	GCTCCAGCATCCACTGGCC	
b52570-interR	CCGCTGACATCGCCCTCTATG	Verification primers for *b52570* deletion
b52570-outerF	CGCTGCAAGCATTGCCGTAC	
b52570-outerR	TTCTCCTTCCGTCACCGGC	
16S rRNA-qF	GTGATAAGCCGAGAGGAAGG	
16S rRNA-qR	CACTGTCACCACCATTGTAG	
c17580-qF	GCAATGCGTATGATGGGCGT	
c17580-qR	GTGCCGCGAGTTCTTTTGACA	
c19200-qF	CCATATTCAGAAGCACCGCG	
c19200-qR	GTTCCAGACCGTGCTTGAGA	
c23720-qF	CTTTCGCTGATGAACGCCAG	
c23720-qR	CTGTCGTAGGAAATGGCGGT	
c31640-qF	GTGCTCCCCTCTTTGTCGAA	Primers used for qRT-PCR
c31640-qR	AGCACGATGAAACCGCAGA	
b52570-qF:	AGTCTGCCTCATCTTTCGCC	
b52570-qR	CTGTGCTTCGCCGTACAATC	
c11920-qF	ATGTCGTCTAATTCCGCCGA	
c11920-qR	TGAGACCCGTCAGCAACAAA	
b47640-qF	CGCTTTGGGATTTCGACGAG	
b47640-qR	TCCGGTAGCCTGACATCGA	

Name	Sequences (5'-3’)	Purpose
c33230$_{EAL}$-qF	ATCACTGAGGGGCTGGAGAT	
c33230$_{EAL}$-qR	AAGTCGTCGAGCCATATCCG	
cuxR-qF	GCAATTTCAGGCTTGGCGAG	
cuxR-qR	GCTGTTGGACGATGAGCAGA	
bgsA-qF	GCGCTATTACCTGCATCTCC	
bgsA-qR	ACAACAGGTTCACGGAGACC	
mcrA-qF	CTGCGGATCGTCACTTCCTG	
mcrA-qR	TGGCGTCGTGAAGGATCTTT	
uppE-qF	CCTACTCGATCCGGCATTGG	
uppE-qR	GATGGTGCGGATCAGGTCTC	
rirA-qF	CTGATGTACTGTGCTGCGAA	
rirA-qR	CTTGAACAGAAACAGCTCGGA	
fliG-qF	AGTTCGAGGACCTGTTCACC	
fliG-qR	GTCAGGCCCTCTTCGAGAAT	
pTc17580-F	TGCCTGATCGATCAATGGTCGC	For construction of pTO-
pTc17580-R	ATGGCAAGCCCTTAAAATTAATTCTCTCTAATGTC	c17580
pTc33230-F	CAAATTCAGCAAAACTTAATGGACCTGAAC	For construction of pTO-
pTc33230-R	GCACCGATCCATTGCGTTTG	c33230
pTc11920-F	GGGCCGGCGGTGGTTTTGAAG	For construction of pTO-
pTc11920-R	GATGAGTCTGCGGACGAGAATTGAAGGGC	c11920
pTc19200-F	GGGCCGGCGGTGGTTTTGAAG	For construction of pTO-
pTc19200-R	GATGAGTCTGCGGACGAGAATTGAAGGGC	c19200
pTc23720-F	TCCTCGATCGCCGCAGCGGC	For construction of pTO-
pTc23720-R	CGGATGCTCCCGGCGGCG	c23720
pTc31640-F	TTCATCGCGCTGCGTTCGG	For construction of pTO-
pTc31640-R	GGATAATCCTCCCTCTTTGCAACTCCAGG	c31640
pTb47640-F	CTAAATGCCCAGGACCACACCGCACC	For construction of pTO-
pTb47640-R	CCTTCAGGACGAGCCGGGCG	b47640
pTb52570-F	GCCGGCCTCCGTTGCCGT	For construction of pTO-
pTb52570-R	CCATGCTGACACGCATCAGAATTTACCGTCATTCAGCG	b52570
pTc06410-F	TCGACTGCCTGGGCAGCAGATTTC	For construction of pTO-
pTc06410-R	GCCGAGCTCCAGGATAGGACATACG	c06410
pTc31480-F	GGTTGGTCTGCTCCAGCTTGAACAAC	For construction of pTO-
pTc31480-R	TTCGCTTCGTTTCACATGTTATACGGCTTGCC	c31480
pTc15850-F	AGCATTGTTTAATGAATTCTTCGTGCG	For construction of pTO-
pTc15850-R	CGACAGAATCCGAAACTCCC	c15850
Cy5-c04750IR-F	GCTGCGTTCCGAGCCGGTGCGCGCTCCGGCCGGGAGCGCATCGCA	
Cy5-c04750IR-R	TGCGATGCGCTCCCGGCCGGAGCGCGCACCGGCTCGGAACGCAGC	
Cy5-c06410IR-F	TCGACTGCCTGGGCAGCAGATTTC	
Cy5-c06410IR-R	GCCGAGCTCCAGGATAGGACATACG	Primers used for EMSA
Cy5-c31480IR-F	GGTTGGTCTGCTCCAGCTTGAACAAC	
Cy5-c31480IR-R	TTCGCTTCGTTTCACATGTTATACGGCTTGCC	
Cy5-c17580IR-F	Cy5-TGCCTGA TCGATCAATGGTCGC	

Name	Sequences (5'-3’)	Purpose
Cy5-c17580IR-R	ATGGCAAGCCCTTAAAATTAATTCTCTCTAATGTC	
Cy5-c33230IR-F	Cy5-CAAATTCAGCAAAACTTAATGGACCTGAAC	
Cy5-c33230IR-R	GCACCGATCCATTGCGTTTG	
Cy5-c11920IR-F	Cy5-GGGCCGGCGGTGGTTTTGAAG	
Cy5-c11920IR-R	GATGAGTCTGCGGACGAGAATTGAAGGGC	
Cy5-c19200IR-F	Cy5-GGGCCGGCGGTGGTTTTGAAG	
Cy5-c19200IR-R	GATGAGTCTGCGGACGAGAATTGAAGGGC	
Cy5-c23720IR-F	Cy5-TCCTCGATCGCCGCAGCGGC	
Cy5-c23720IR-R	CGGATGCTCCCGGCGGCG	
Cy5-c31640IR-F	Cy5-TTCATCGCGCTGCGTTCGG	
Cy5-c31640IR-R	GGATAATCCTCCCTCTTTGCAACTCCAGG	
Cy5-b47640IR-F	Cy5-CTAAATGCCCAGGACCACACCGCACC	
Cy5-b47640IR-R	CCTTCAGGACGAGCCGGGCG	
Cy5-c15850IR-F	AGCATTGTTTAATGAATTCTTCGTGCG	
Cy5-c15850IR-R	CGACAGAATCCGAAACTCCC	
Cy5-b52570IR-F	Cy5-GCCGGCCTCCGTTGCCGT	
Cy5-b52570IR-R	CCATGCTGACACGCATCAGAATTTACCGTCATTCAGCG	
